# Maxillary First Premolars’ Internal Morphology: A Systematic Review and Meta-Analysis

**DOI:** 10.3390/dj13110510

**Published:** 2025-11-03

**Authors:** Thomas Gerhard Wolf, Dilara Sare Ulugöl, Richard Johannes Wierichs, Agnes Klara Maria Holtkamp, Gianrico Spagnuolo, David Donnermeyer, Andrea Lisa Waber

**Affiliations:** 1 Department of Restorative, Preventive and Pediatric Dentistry, School of Dental Medicine, University of Bern, CH-3010 Bern, Switzerland; 2 Department of Periodontology and Operative Dentistry, University Medical Center of the Johannes Gutenberg-University Mainz, D-55131 Mainz, Germany; 3 Department of Periodontology, Operative and Preventive Dentistry, University of Bonn, D-53111 Bonn, Germany; 4 Department of Prosthodontics, Geriatric Dentistry and Craniomandibular Disorders, Charité—Universitätsmedizin Berlin, D-14197 Berlin, Germany; 5 Department of Neurosciences, Reproductive and Odontostomatological Sciences, University of Naples “Federico II”, I-80131 Napoli, Italy

**Keywords:** internal morphology, meta-analysis, maxillary first premolars, root canal configuration, systematic review, sex differences

## Abstract

**Objectives:** This systematic review analyzed the root canal morphology and configuration (RCC) of maxillary first premolars (Mx1Ps) and sex-specific differences based on existing literature. **Methods:** Registered in PROSPERO (CRD42023394460) and following PRISMA guidelines, systematic searches were conducted in five databases (Cochrane, Embase, LILACS, Scopus, MEDLINE via PubMed) using predefined MeSH terms. Additional studies were identified through cross-referencing. Studies on Mx1P RCCs were included, assessed using the AQUA tool. Data extraction focused on RCC prevalence, sex differences, root variations, and examination methods. **Results:** Of 865 studies, 86 were included, analyzing 31,325 teeth. The most common RCCs were 2-2-2/2 (IV, frequencies between 0.6–80.5%) and 1-1-1/1 (I, 1.1–72.0%). Mx1Ps primarily had two roots (7.1–96.2%) or one root (3.8–93.2%), with three-rooted variants being rare (0.4–6.5%). Males more frequently exhibited two- or three-rooted Mx1Ps with RCCs like 2-2-2/2 (IV; OR = 1.39 [1.22, 1.58]), and 1-1-3/3 (VIII; OR = 2.22 [1.59, 3.11]). Females showed higher frequencies of RCCs like 1-1-1/1 (I; OR = 0.71 [0.53, 0.96]), 2-2-1/1 (II; OR = 0.66 [0.57, 0.77]), 1-2-1/1 (III; OR = 0.70 [0.59, 0.83]), and 1-1-2/2 (V; OR = 0.81 [0.70, 0.95]). **Conclusions:** Mx1Ps predominantly have two roots and a 2-2-2/2 (IV) RCC. CBCT was the most used method, followed by staining and clearing. Clinicians should consider sex-specific and morphological variations.

## 1. Introduction

The primary goal of clinical endodontic treatment is to thoroughly eliminate bacteria, prepare the root canal system through mechanical shaping, chemical cleaning, and subsequent obturation with filling material. However, untreated root canals often lead to treatment failure, primarily because they are not identified by the clinician [1]. Insufficient knowledge of root canal anatomy remains one of the key reasons for endodontic failure [2]. To minimize the risk of missing canals and errors during access cavity preparation, cleaning, shaping, or obturation, a comprehensive understanding of root canal morphology is essential [3], especially regarding sex-specific and morphological differences in the maxillary first premolars, which are important for an accurate diagnosis and successful root canal treatment. Over the years, various methods have been employed to study the root canal anatomy of maxillary first premolars. Earlier techniques included radiography [2,4,5,6,7,8], grinding [9], staining and clearing [2,10,11,12,13,14,15,16,17,18,19,20,21,22,23], microscopy [9,11,12,13,14,18,20,23], or cross-sectional analysis [2,5,8,22]. Additionally, one study used polyester casting resin combined with red dye for anatomical evaluation [24]. In recent decades, cone-beam computed tomography (CBCT) [25,26,27,28,29,30,31,32,33,34,35,36,37,38,39,40,41,42,43,44,45,46,47,48,49,50,51,52,53,54,55,56] has provided more detailed insights. While CBCT enables retrospective in vivo anatomical investigations [57,58,59,60,61,62,63,64,65,66,67,68,69,70,71,72,73,74,75,76,77,78,79,80,81,82,83,84,85,86], micro-CT remains restricted to ex vivo studies [87,88]. The results of these studies are frequently reported using different classification systems, including Vertucci’s system [1] and that of Weine et al. [89]. For more complex root canal systems, classification methods such as those proposed by Sert et al. [13], Briseño-Marroquín et al. [3] or Ahmed et al. [90] have been utilized. Briseño-Marroquín et al. [3], for example, describe the root canal system in four digits: the first three digits represent the morphology of the coronal, middle, and apical thirds of the root, while the last digit indicates the number of main apical foramina. The maxillary first premolar is often characterized by two roots and a predominant root canal configuration (RCC) of 2-2-2/2 (Type IV according to Vertucci and Type III according to Weine). However, significant variations in the number of roots and RCCs have been reported in the literature [2].

The aim of this systematic review was to analyze the internal root canal morphology of Mx1Ps and to investigate sex-related differences using meta-analysis.

## 2. Materials and Methods

This review was registered in the International Prospective Register of Systematic Reviews (PROSPERO) system of the National Institute of Health Research of the Centre for Reviews and Dissemination of the University of York (United Kingdom) (CRD42023394460). This systematic review follows the PRISMA guidelines (Preferred Reporting Items for Systematic Reviews and Meta-Analyses) (Supplementary Materials File S1) [84,91]. This systematic review was structured according to the PICO framework: studies that examined the root canal morphology and configuration of the first premolars of the human maxilla (population) using various methods such as irrigation, X-rays, CBCT, or micro-CT (intervention) without a specific comparison (comparison) were analyzed in terms of the frequency and characteristics of root canal configurations, including the number of roots, physiological foramina, accessory canals, anastomoses, and dimensions of physiological foramina (outcome). A literature search was conducted in five electronic databases (MEDLINE via PubMed, Embase, Scopus, LILACS, and Cochrane Database) on the RCC of the maxillary first premolar. The search was conducted until September 2025 using the following Medical Subject Heading (MeSH) terms and search terms (search string): (root canal configuration OR root canal system OR root canal morphology) AND (morphology OR anatomy) AND (maxillary first premolar OR maxillary premolars). The identical search string was used without the application terms or filters across all databases (MEDLINE via PubMed, Embase, Scopus, LILACS, and the Cochrane database). Further relevant studies were added to the list through cross-referencing and handsearching. Thus, further papers were looked for manually by screening reference lists or citations of articles already found. Duplicates were removed by using EndNote version 20.6 (Clarivate, Philadelphia, PA, USA) and comparing the articles retained from the databases. The remaining articles were screened for relevance based on title and abstract by two independent reviewers (D.S.U., A.L.W.). Articles that were irrelevant to the topic were excluded. The remaining articles that met the inclusion criteria were viewed in full text and were examined again by the same two independent reviewers (D.S.U., A.L.W.).

### 2.1. Inclusion and Exclusion Criteria

There were no language restrictions. Case reports, reviews, and papers addressing other morphological research questions than RCC were excluded. Articles in which the teeth were not clearly identified as maxillary first premolars or in which certain information, such as the total number of teeth examined or the population studied, was missing, were also excluded. Randomized controlled trials, comparative studies, evaluation and validation studies, and cross-sectional studies of any population and of all age groups were included.

### 2.2. Data Collection and Synthesis

The authors of the papers were identified, along with the date of publication, the population studied, and the number of teeth, the methods used in the studies for analysis, and the data obtained regarding the number of roots and the RCC. The information was summarized in tables. The classifications of Vertucci [1], Weine et al. [89] and Briseño-Marroquín et al. [3] were used to present these results. In recent years, the system proposed by Ahmed et al. [90] has been increasingly utilized in the literature. In this paper, RCC was not presented using this system, as it is difficult to correlate older classification systems, such as the one proposed by Vertucci [1], which has been used in a great number of the articles included in this review. The Review Manager software (RevMan version 5.4 software, Cochrane Collaboration, Copenhagen, Denmark, 2014) was used for statistical analysis and meta-analyses. The odds ratio was calculated to determine the effect size. The I2 statistic was determined to quantify the degree of variation due to heterogeneity instead of chance [92]. Depending on the heterogeneity (I2 < 35% for low heterogeneity, fixed effects and I2 > 35% for high heterogeneity, random effects), a meta-analysis was carried out. The primary measures of effect were the odds ratio and 95% confidence intervals (95% CI), comparing different RCC geographic regions and patients’ sex for studies with dual outcome data. Using a *p*-value of 0.05 or less indicates statistical significance.

### 2.3. Quality Assessment and Risk of Bias

We assessed the quality of the studies using the quality assessment tools provided by the National Heart, Lung and Blood Institute (https://www.nhlbi.nih.gov/health-topics/study-quality-assessment-tools, last accessed on 23 June 2025) (Supplementary Materials File S2). We used the Anatomical Quality Assessment (AQUA) tool to assess the risk of bias in the studies [93] (Supplementary Materials File S3). The AQUA tool uses five areas with a total of twenty questions to determine whether a study is at risk of error. The five areas are as follows: (1) aim and characteristics of the teeth examined, (2) study design, (3) description of the research methods used, (4) illustration of anatomy and (5) reporting of the results. Each question is answered with “yes” or “no”. Depending on the answers, the work can ultimately be classified as “at high risk of bias” or “at low risk of bias”. If all questions for a section are answered with “yes”, then the risk of bias is “low”. The quality of the studies included was examined by two independent reviewers (D.S.U., A.L.W.). In the event of a disagreement, a third reviewer (T.G.W.) was consulted.

## 3. Results

**Figure 1:**
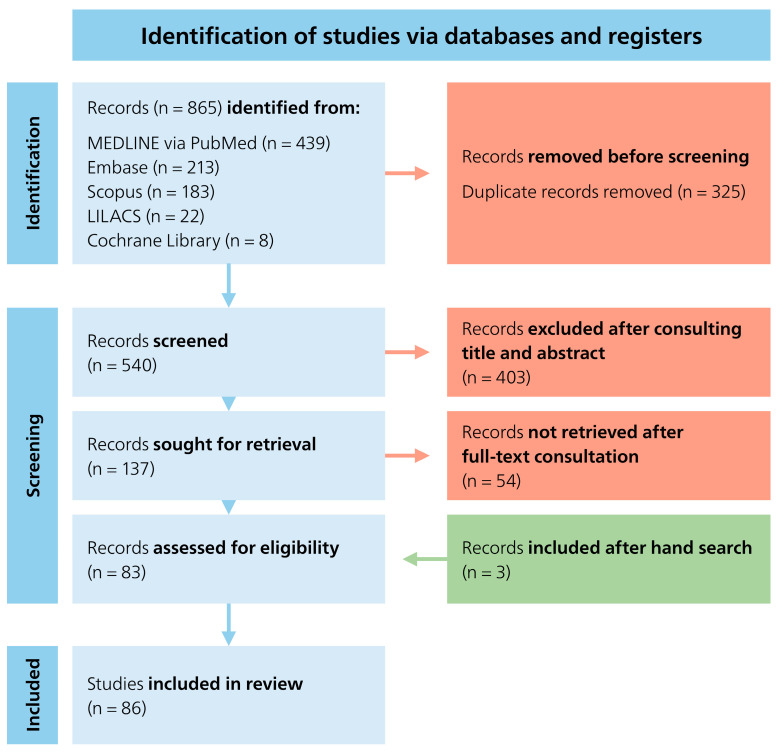
PRISMA flowchart [94].

The literature search in the five databases (MEDLINE via PubMed, Embase, Scopus, LILACS and Cochrane Database) yielded a total of 865 articles. After the duplicates were removed, 540 articles remained, which were checked for inclusion and exclusion criteria based on the title and abstract. In this process, 403 articles were excluded because they did not fit the topic. The remaining 137 articles that fulfilled the inclusion criteria were reviewed in full text. After analyzing the full-text articles and handsearching, three additional articles were added. Thus, a total of 86 articles were included in this systematic review (Figure 1).

Table S1 (Supplementary Materials File S4) summarizes all the results and details of the articles listed, such as authors, date of publication, population studied, and research method used.

The RCC and the number of roots were presented in Table S2 (Supplementary Materials File S5) based on the classification systems of Vertucci [1], Weine et al. [89], and Briseño-Marroquìn et al. [3].

The total number of teeth examined was 31,325. The articles examined the differences in root canal morphology of the maxillary first premolar in different populations and between males and females. Some studies also compared the maxillary right first premolar [14] to the maxillary left first premolar [24]. Two studies compared root canal morphology in the same population within different age groups [36,74]. The most used examination method was CBCT imaging, followed by staining and clearing of the canals. Analysis of RCC using a microscope, radiographs, cross sections, micro-CT and grinding of teeth was less frequently performed. More recent studies favor CBCT imaging. Only two of the included studies used micro-CT to visualize root canal morphology [87,88].

### 3.1. Number of Roots

The results show that most maxillary first premolars have two roots, with a frequency between 7.1 and 96.2% [2,5,6,8,10,12,14,16,17,18,27,28,31,32,34,38,39,40,42,43,45,46,48,49,52,54,55,56,57,60,61,62,63,65,66,68,69,73,74,75,77,79,81,83,86]. The first premolars in the upper jaw with one root showed a proportion between 3.8 and 93.2% [15,19,20,21,23,25,30,33,35,38,41,44,47,50,51,59,64,67,71,72,78,80,82,84,85]. Three roots were observed with a much lower frequency between 0.3 and 11.7% [2,5,8,10,12,14,15,17,18,19,20,21,25,27,28,30,31,32,33,34,35,37,38,40,41,42,43,44,45,46,47,48,49,51,52,54,56,57,60,61,63,64,65,66,69,70,71,73,74,75,78,79,80,81,83,84,86].

### 3.2. Root Canal Configuration (RCC)

The most frequently observed RCC was type 2-2-2/2 according to Briseño-Marroquín (type III according to Weine and type IV according to Vertucci), with a frequency of 0.6–80.5% [2,4,5,6,7,8,9,10,11,12,13,14,15,16,17,19,21,23,24,25,26,27,29,30,31,32,33,34,35,36,37,38,40,41,42,43,45,46,47,48,49,51,52,55,56,57,58,59,61,62,65,67,71,73,74,75,76,77,79,81,82,83,84,85,86,87,88]. The second most frequently observed classification was 1-1-1/1 (type I according to Weine and type I according to Vertucci) with a frequency of 1.1–72% [28,39,54,63,72,78,80]. The RCC 2-2-1/1 (type II according to Weine and type II according to Vertucci) was reported as the most common configuration in just one article, with a frequency of 23.8% [44]. 1-1-2/2 (Weine type IV and Vertucci type V) was reported as the most common RCC in four articles, with a frequency of up to 58.5% [22,50,60,64]. 3-3-3/3 (type VIII according to Vertucci) was observed with a frequency of only up to 11.7%.

### 3.3. Comparative Studies

This review also includes comparative studies. The differences between women and men [13,17,23,26,31,34,37,43,45,47,48,52,59,61,62,64,65,71,72,74,75,76,79,81,82,83,84,85,86], the left and right first premolars in the maxilla [26,31,45,47,52,56,59,62,65,67,69,70,71,75] and between different populations [15,38,60,83,85] were investigated. Two studies compared RCCs within different age groups [36,74,79,81,86].

**Figure 2:**
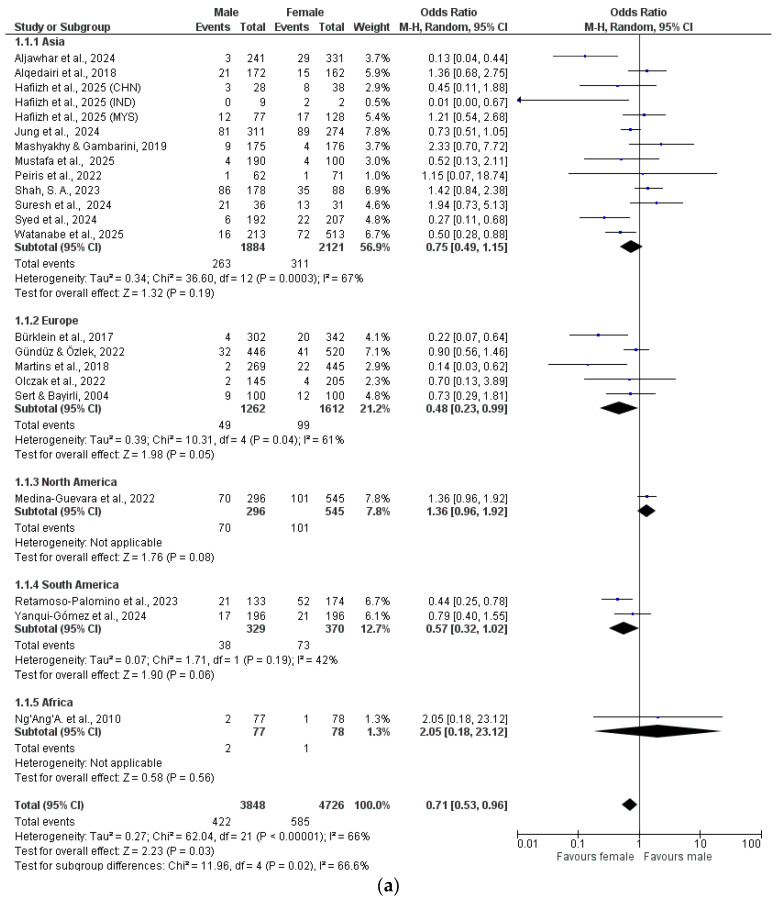

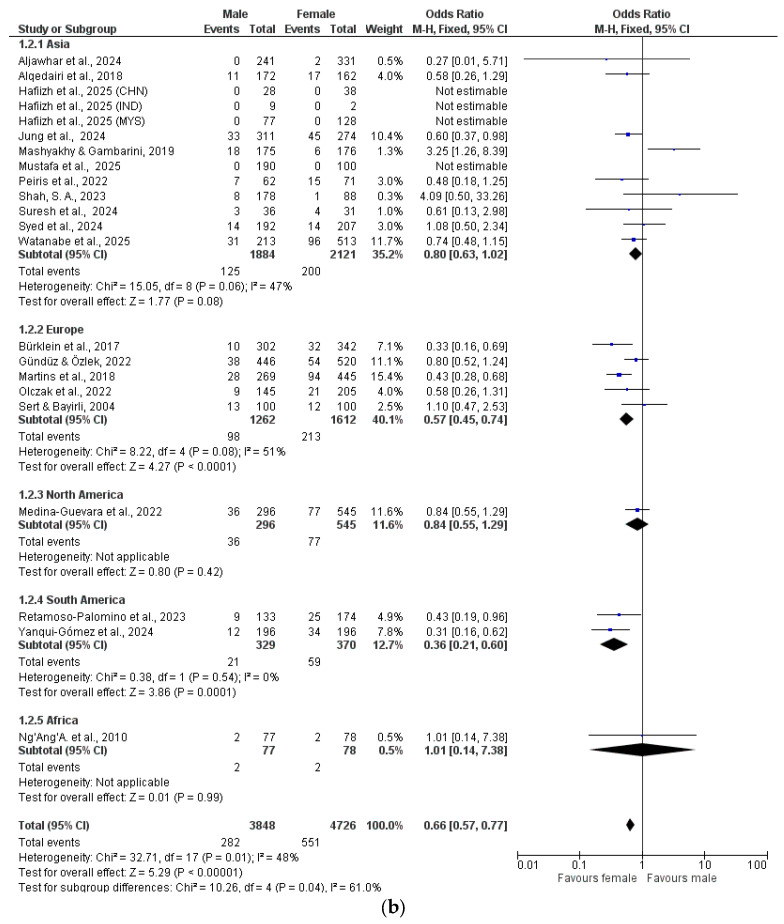

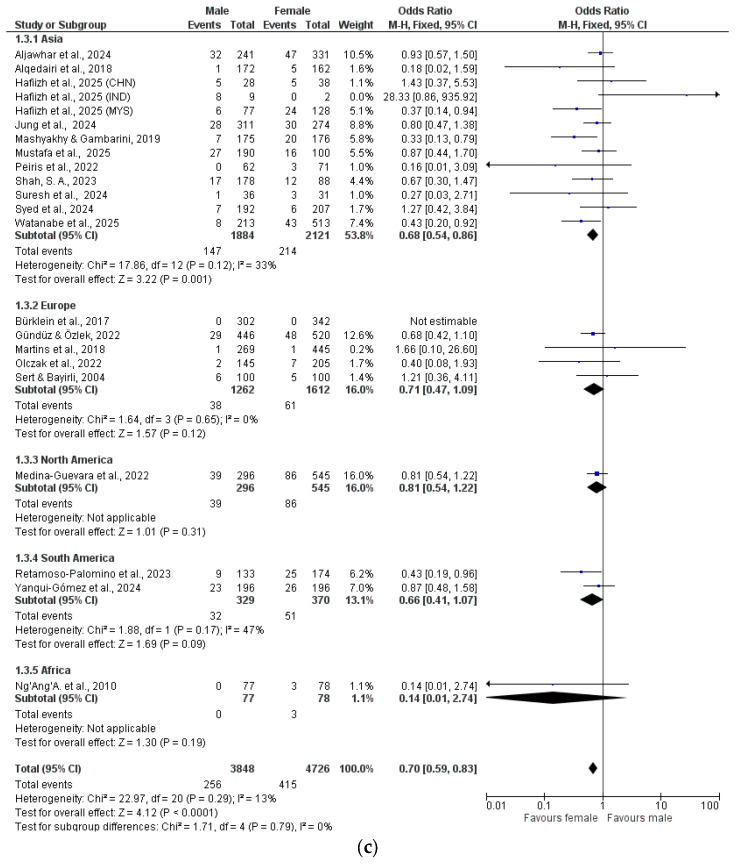

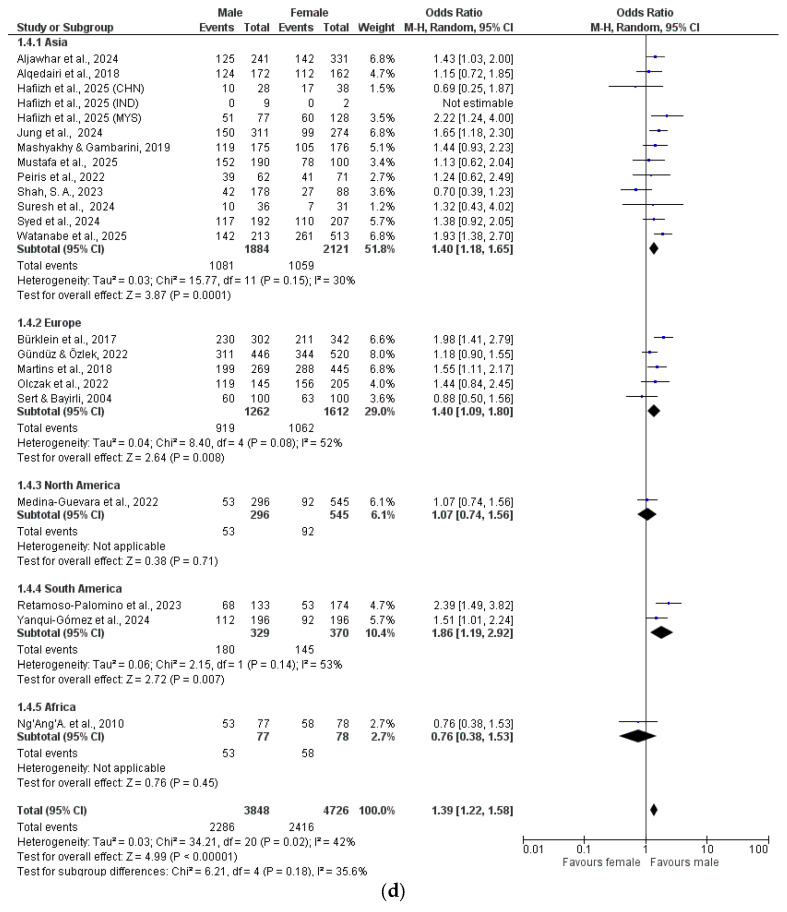

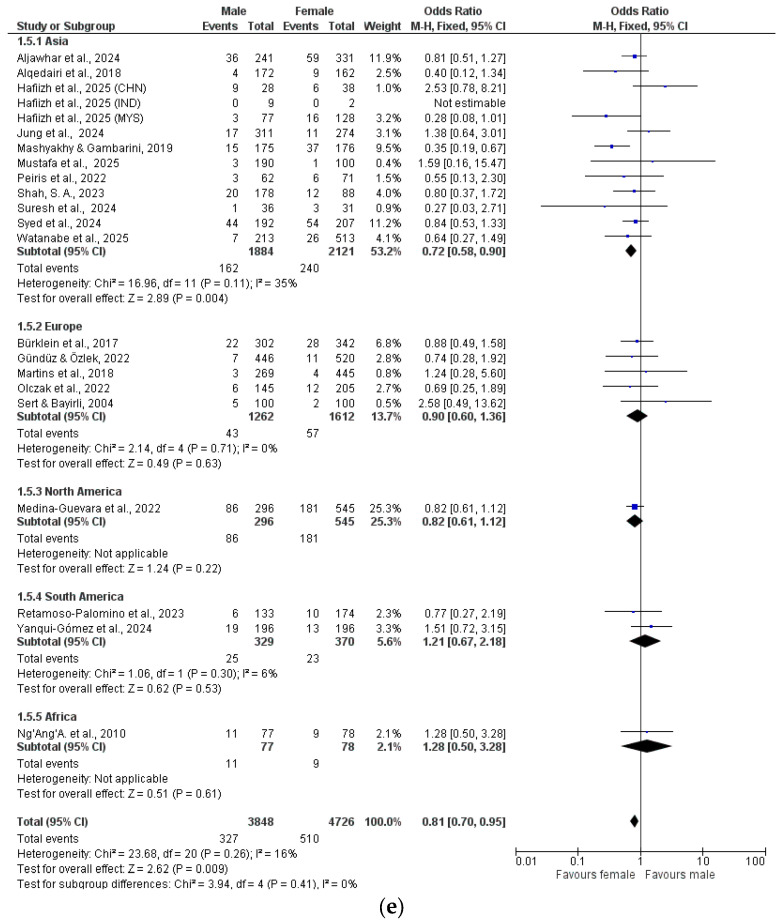

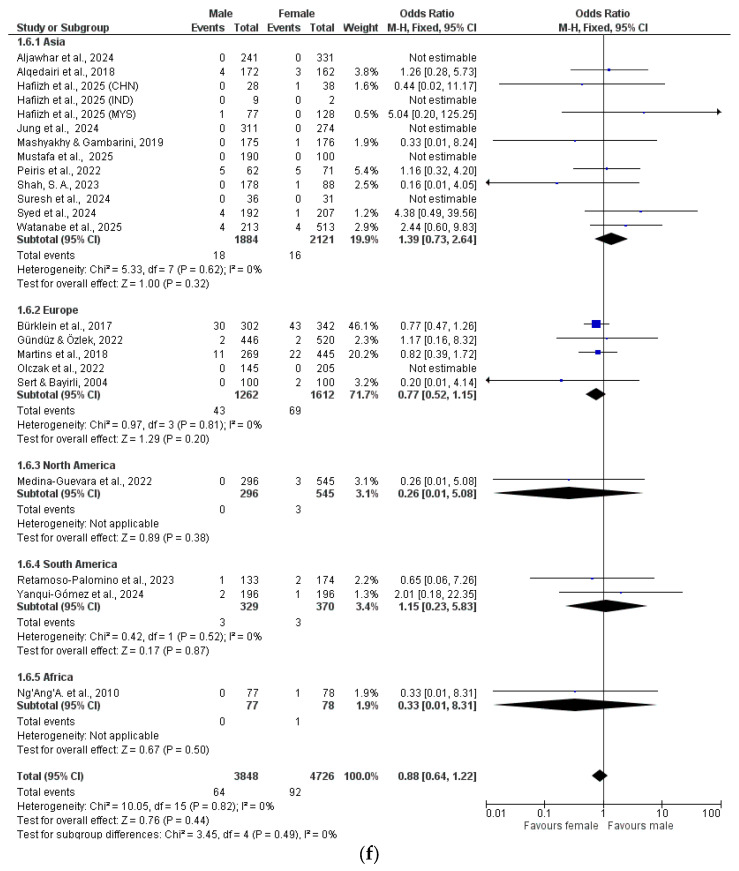

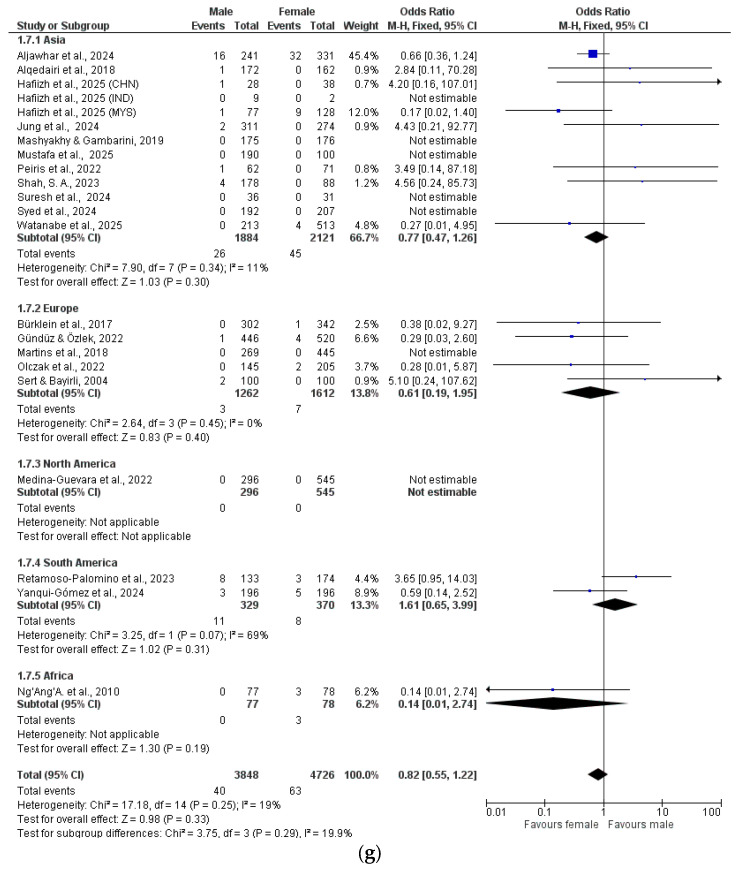

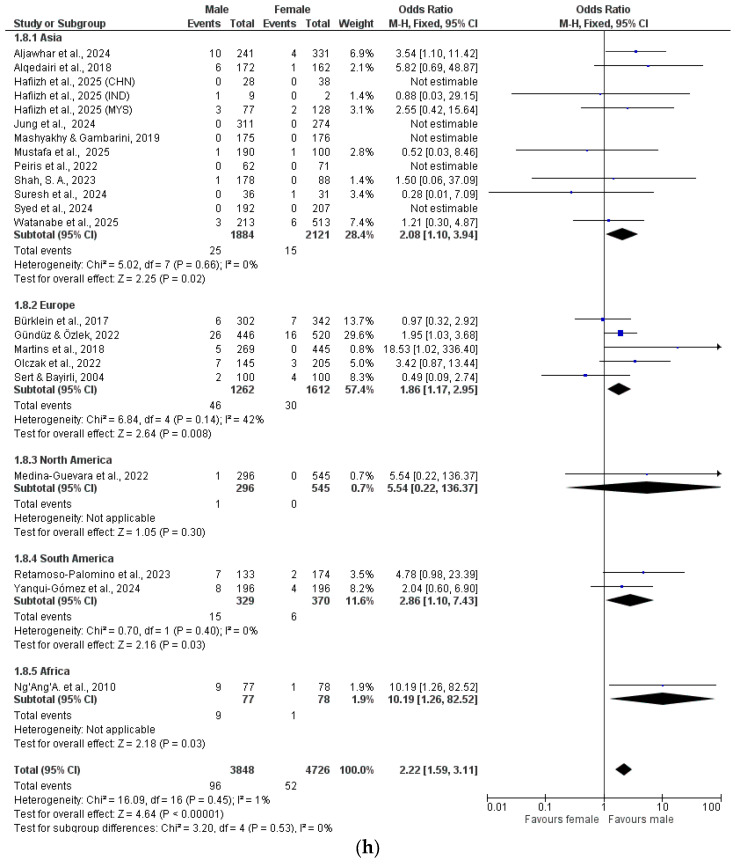
(**a**) Root canal configuration 1-1-1/1 (Vertucci I). (**b**) Root canal configuration 2-2-1/1 (Vertucci II). (**c**) Root canal configuration 1-2-1/1 (Vertucci III). (**d**) Root canal configuration 2-2-2/2 (Vertucci IV). (**e**) Root canal configuration 1-1-2/2 (Vertucci V). (**f**) Root canal configuration 2-1-2/2 (Vertucci VI). (**g**) Root canal configuration 1-2-1/2 (Vertucci VII). (**h**) Root canal configuration 1-1-3/3 (Vertucci VIII). [13,17,23,31,34,37,43,61,64,65,71,72,74,76,79,80,81,82,85,86].

The data were analyzed by meta-analysis and grouped by geographical location (Asia, Europe, North America, South America, Africa) (Figure 2a–h).

## 4. Discussion

The aim of this paper was to summarize the possible variations in RCC of the maxillary first premolar in the form of a systematic review and to provide the practitioner with an understanding and knowledge of what to expect during a root canal treatment.

### 4.1. Number of Roots and Root Canal Configuration (RCC)

This systematic review shows that the first maxillary premolar usually has two roots. The lowest reported frequency was 7.1% [72], while the highest frequency was 96.2% [60]. However, many studies reported only one root with frequencies between 3.8% and 93.8%. The lowest and highest frequencies were again reported by Alnaqbi et al. [60] and Shah [72], respectively. Three roots were found in many studies, but with a lower frequency of between 0.4–6.5% [17,69]. A RCC of 2-2-2/2 (type IV according to Vertucci and type II according to Weine) was the most frequently reported RCC with a frequency of up to 80.5% [69]. In teeth with two roots, the frequency of the configuration can be as high as 99.6% [47]. However, in some of the included studies, for example, by Bulut et al., the frequency was very low at 1.9% [28]. The range of results is thus very wide, and the values are heterogeneous. Several studies [28,39,54,63,72] report that a RCC of 1-1-1/1 is the most common RCC in their samples. The frequency ranges between 45.4 and 72.0%, whereby it was striking that three of the five studies were conducted on a Pakistani population. The RCCs 2-2-1/2 (type II according to Vertucci) and 1-1-2/2 (type V according to Vertucci) were reported as the most common RCC in only five studies. First maxillary premolars with three roots and the configuration 1-1-3/3 were even described as being present in 100% of cases in various populations [25,27,35,40,47,48]. Only one study [33] found other, not further classified, RCCs in three-rooted teeth with a frequency of 14.3%. All other RCCs, such as 1-2-1/1, 2-1-2/2 and 1-2-1/2, were also reported in most papers but occur less frequently.

### 4.2. Differences Between Women and Men

This systematic review examined sex-specific differences in the anatomy of the first maxillary premolar, with 20 studies included in the meta-analysis. Except for the studies by Sert et al. [13], Ng’Ang’A et al. [17] and Peiris et al. [23], all were conducted using CBCT. The included studies show that maxillary first premolars with only one root are more common in women [17,23,31,34,37,43,61,74,85,86]. The frequency is up to 100% [23]. In contrast, maxillary first premolars with two or three roots are more common in men [17,23,31,34,37,43,61,65,74,80,83,84] with a frequency of 62.1% [37] and 83.1% [17], respectively. Men also more often had additional RCCs that Vertucci could not define [13,23,37,43,79,86], with Martins et al. [37] reporting 20%. A significantly higher number of the RCCs 1-1-1/1 (Vertucci Type I), 2-2-1/1 (Vertucci Type II), 1-2-1/1 (Vertucci Type III), 1-1-2/2 (Vertucci Type V) and 2-1-2-/2 (Vertucci Type VI) and (Vertucci Type VII) were observed in females. However, the RCCs 2-2-2/2 (Vertucci Type IV) and 1-1-3/3 (Vertucci Type VIII) were observed with a higher number in males. For each RCC, sex-specific differences were observed. The age of the patients was not reported in all the studies included; therefore, the age was not included in the meta-analysis either.

### 4.3. Sample Size and Research Methods

The number of teeth examined in the studies included in this work varies strongly. The sample sizes varied between 24 [6] and 1387 [35] examined teeth. Most studies investigated more than 100 teeth. Different techniques were used to examine the anatomy of the samples, for example, X-ray [2,4,5,6,7,8], grinding [9], staining and clearing [2,10,11,12,13,14,15,16,17,18,19,20,21,22,23], microscopy [9,11,12,13,14,18,20,23], cross-sectioning [2,5,8,22] and micro-CT [87,88]. CBCT imaging was used in most of the studies examined in this systematic review [25,26,27,28,29,30,31,32,33,34,35,36,37,38,39,40,41,42,43,44,45,46,47,48,49,50,51,52,53,54,55,56,57,58,59,60,61,62,63,64,65,66,67,68,69,70,71,72,73,74,75,76,77,78,79,80,81,82,83,84,85,86]. Although micro-CT is considered the gold standard in terms of accuracy and resolution, it is limited to ex vivo application and thus only used in research. It should also be mentioned that scanning and reconstructing a single tooth from the sample is very time-consuming, depending on the resolution, and that artifacts, e.g., due to drying of the teeth, can lead to misinterpretations or errors in the analysis [95,96,97,98]. The other methods mentioned, such as X-rays, grinding, staining and clearing, the use of a microscope and the examination of cross-sections, now appear to have been largely replaced by CBCT imaging as a modern three-dimensional technique in both research and practice. As these previous techniques are used less frequently in more recent studies, especially for issues that cannot be resolved with two-dimensional techniques, three-dimensional techniques are intended to provide additional information. The most popular way of describing RCCs was the method according to Vertucci [1]. Only three papers used the methods of Weine et al. [2,5,22], and one paper used the method of Briseño-Marroquín et al. [3].

### 4.4. Strengths and Limitations

A limitation of the present work arises from the fact that, due to the diversity of the studies included and the different research methods, the results must be considered with caution. The studies examine different populations, ethnic groups and various countries and often work with age groups that are not clearly defined, and information on sex, age and position in the maxilla (right, left) is often missing. The sample sizes examined also differ significantly, with most studies not calculating the sample size. Although several classification systems for root canal configuration have been proposed over the years, we focused our analysis on the three most used and widely accepted systems, which may be considered a limitation of the search strategy. The strength of this work lies in the large amount of literature from both retrospective studies and in vivo studies that could be included and summarized in this work. Teeth from 32 different populations were examined, so the results can be better applied to the general population of the respective geographic regions and are useful for clinical practice.

## 5. Conclusions

The internal morphology in teeth can be examined using various methods, with CBCT being the most widely utilized. Maxillary first premolars predominantly exhibit two roots, with reported frequencies ranging from 7.1–96.2%. The most common root canal configuration (RCC) is 2-2-2/2 (Vertucci type IV, Weine type III), followed by 1-1-1/1 (Vertucci type I, Weine type I). Other observed RCCs include 1-2-1/1 (Vertucci type III), 2-1-2/2 (Vertucci type VI), and 1-2-1/2 (Vertucci type VII).

Sex differences were evident: male patients more frequently presented with two or three-rooted maxillary first premolars, with RCCs such as 2-2-2/2 (Vertucci type IV) and 1-1-3/3 (Vertucci type VIII) observed more often. In contrast, females exhibited higher frequencies of RCCs like 1-1-1/1 (Vertucci type I), 2-2-1/1 (Vertucci type II), 1-2-1/1 (Vertucci type III), 1-1-2/2 (Vertucci type V), and 2-1-2/2 (Vertucci type VI).

This systematic review and meta-analysis provide valuable insights into the internal morphology of maxillary first premolars, aiming to help clinicians in optimizing treatment strategies. However, findings should be interpreted cautiously due to inherent limitations. Variations in RCCs, along with patient-specific factors such as ethnicity, sex, and age, must be considered to minimize errors and avoid iatrogenic complications during root canal treatments.

## Data Availability

All relevant data generated or analyzed in this study are provided within the manuscript and Supplementary Materials due to data protection regulations. For any additional information, the corresponding author can be contacted upon reasonable request.

## Supplementary Materials

The following supporting information can be downloaded at: https://www.mdpi.com/article/10.3390/dj13110510/s1, File S1: PRISMA checklist; File S2: Quality assessment; File S3: AQUA tool evaluation; File S4: Table S1. Characteristics of the included studies of Mx1Ps categorized by authors, study type, teeth investigated, methodology, and country of origin; File S5: Table S2. RCCs depicted according to the classifications.

## References

[1] Vertucci F.J. (1984). Root canal anatomy of the human permanent teeth. Oral Surg. Oral Med. Oral Pathol..

[2] Özcan E., Çolak H., Hamidi M.M. (2012). Root and canal morphology of maxillary first premolars in a Turkish population. J. Dent. Sci..

[3] Briseño-Marroquín B., Paqué F., Maier K., Willershausen B., Wolf T.G. (2015). Root canal morphology and configuration of 179 maxillary first molars by means of micro-computed tomography: An ex vivo study. J. Endod..

[4] Pineda F., Kuttler Y. (1972). Mesiodistal and buccolingual roentgenographic investigation of 7,275 root canals. Oral Surg. Oral Med. Oral Pathol..

[5] Atieh M.A. (2008). Root and canal morphology of maxillary first premolars in a Saudi population. J. Contemp. Dent. Pract..

[6] Agholor C.N., Sede M.A. (2021). An evaluation of root and canal morphology of maxillary premolars in a Nigerian tertiary hospital: An in-vivo study. Ghana Dent. J..

[7] Qiao X., Xu T., Chen L., Yang D. (2021). Analysis of root canal curvature and root canal morphology of maxillary posterior teeth in Guizhou, China. Med. Sci. Monit..

[8] Faraj B.M., Abdulrahman M.S., Faris T.M. (2022). Visual inspection of root patterns and radiographic estimation of its canal configurations by confirmation using sectioning method: An ex vivo study on maxillary first premolar teeth. BMC Oral Health.

[9] Green D. (1973). Double canals in single roots. Oral Surg. Oral Med. Oral Pathol..

[10] Vertucci F.J., Gegauff A. (1979). Root canal morphology of the maxillary first premolar. J. Am. Dent. Assoc..

[11] Calişkan M.K., Pehlivan Y., Sepetçioglu F., Türkün M., Tuncer S.S. (1995). Root canal morphology of human permanent teeth in a Turkish population. J. Endod..

[12] Kartal N., Ozçelik B., Cimilli H. (1998). Root canal morphology of maxillary premolars. J. Endod..

[13] Sert S., Bayirli G.S. (2004). Evaluation of the root canal configurations of the mandibular and maxillary permanent teeth by gender in the Turkish population. J. Endod..

[14] Awawdeh L., Abdullah H., Al-Qudah A. (2008). Root form and canal morphology of Jordanian maxillary first premolars. J. Endod..

[15] Peiris R. (2008). Root and canal morphology of human permanent teeth in a Sri Lankan and Japanese population. Anthropol. Sci..

[16] Weng X.-L., Yu S.-B., Zhao S.-L., Wang H.-G., Mu T., Tang R.-Y., Zhou X.-D. (2009). Root canal morphology of permanent maxillary teeth in the Han nationality in Chinese Guanzhong area: A new modified root canal staining technique. J. Endod..

[17] Ng’ang’a R.N., Masiga M.A., Maina S.W. (2010). Internal root morphology of the maxillary first premolars in Kenyans of African descent. East Afr. Med. J..

[18] Neelakantan P., Subbarao C., Ahuja R., Subbarao C.V. (2011). Root and canal morphology of Indian maxillary premolars by a modified root canal staining technique. Odontology.

[19] Gupta S., Sinha D.J., Gowhar O., Tyagi S.P., Singh N.N., Gupta S. (2015). Root and canal morphology of maxillary first premolar teeth in north Indian population using clearing technique: An in vitro study. J. Conserv. Dent..

[20] Dinakar C., Shetty U.A., Salian V.V., Shetty P. (2018). Root canal morphology of maxillary first premolars using the clearing technique in a South Indian population: An in vitro study. Int. J. Appl. Basic Med. Res..

[21] Senan E.M., Alhadainy H.A., Genaid T.M., Madfa A.A. (2018). Root form and canal morphology of maxillary first premolars of a Yemeni population. BMC Oral Health.

[22] Khattak M.A., Ijaz F., Ahmad N., Arbab S., Khattak I., Khattak Y.J. (2022). Root canal configuration of maxillary first premolar teeth in a subpopulation of Peshawar using tooth cross-sectioning method: An in vitro study. J. Ayub Med. Coll. Abbottabad.

[23] Peiris R., Arambawatta K., Pitakotuwage N. (2022). Root and canal morphology of maxillary premolars and their relationship with the crown morphology. J. Oral Biosci..

[24] Carns E.J., Skidmore A.E. (1973). Configurations and deviations of root canals of maxillary first premolars. Oral Surg. Oral Med. Oral Pathol..

[25] Tian Y.Y., Guo B., Zhang R., Yu X., Wang H., Hu T., Dummer P.M.H. (2012). Root and canal morphology of maxillary first premolars in a Chinese subpopulation evaluated using cone-beam computed tomography. Int. Endod. J..

[26] Ok E., Altunsoy M., Nur B.G., Aglarci O.S., Çolak M., Güngör E. (2014). A cone-beam computed tomography study of root canal morphology of maxillary and mandibular premolars in a Turkish population. Acta Odontol. Scand..

[27] Abella F., Teixidó L.M., Patel S., Sosa F., Duran-Sindreu F., Roig M. (2015). Cone-beam computed tomography analysis of the root canal morphology of maxillary first and second premolars in a Spanish population. J. Endod..

[28] Bulut D.G., Kose E., Ozcan G., Sekerci A.E., Canger E.M., Sisman Y. (2015). Evaluation of root morphology and root canal configuration of premolars in Turkish individuals using cone-beam computed tomography. Eur. J. Dent..

[29] Felsypremila G., Vinothkumar T.S., Kandaswamy D. (2015). Anatomic symmetry of root and root canal morphology of posterior teeth in an Indian subpopulation using cone-beam computed tomography: A retrospective study. Eur. J. Dent..

[30] Celikten B., Orhan K., Aksoy U., Tufenkci P., Kalender A., Basmaci F., Dabaj P. (2016). Cone-beam CT evaluation of root canal morphology of maxillary and mandibular premolars in a Turkish Cypriot population. BDJ Open.

[31] Bürklein S., Heck R., Schäfer E. (2017). Evaluation of the root canal anatomy of maxillary and mandibular premolars in a selected German population using cone-beam computed tomographic data. J. Endod..

[32] Martins J.N.R., Marques D., Mata A., Caramês J. (2017). Root and root canal morphology of the permanent dentition in a Caucasian population: A cone-beam computed tomography study. Int. Endod. J..

[33] Shi Z.Y., Hu N., Shi X.W., Dong X.X., Ou L., Cao J.K. (2017). Root canal morphology of maxillary premolars among the elderly. Chin. Med. J..

[34] Alqedairi A., Alfawaz H., Al-Dahman Y., Alnassar F., Al-Jebaly A., Alsubait S. (2018). Cone-beam computed tomographic evaluation of root canal morphology of maxillary premolars in a Saudi population. Biomed. Res. Int..

[35] Li Y.H., Bao S.J., Yang X.W., Tian X.M., Wei B., Zheng Y.L. (2018). Symmetry of root anatomy and root canal morphology in maxillary premolars analyzed using cone-beam computed tomography. Arch. Oral Biol..

[36] Martins J.N.R., Ordinola-Zapata R., Marques D., Francisco H., Caramês J. (2018). Differences in root canal system configuration in human permanent teeth within different age groups. Int. Endod. J..

[37] Martins J.N.R., Marques D., Francisco H., Caramês J. (2018). Gender influence on the number of roots and root canal system configuration in human permanent teeth of a Portuguese subpopulation. Quintessence Int..

[38] Martins J.N.R., Gu Y., Marques D., Francisco H., Caramês J. (2018). Differences on the root and root canal morphologies between Asian and White ethnic groups analyzed by cone-beam computed tomography. J. Endod..

[39] Nazeer M.R., Khan F.R., Ghafoor R. (2018). Evaluation of root morphology and canal configuration of maxillary premolars in a sample of Pakistani population by using cone beam computed tomography. J. Pak. Med. Assoc..

[40] de Lima C.O., de Souza L.C., Devito K.L., do Prado M., Campos C.N. (2019). Evaluation of root canal morphology of maxillary premolars: A cone-beam computed tomography study. Aust. Endod. J..

[41] Liu X., Gao M., Ruan J., Lu Q. (2019). Root canal anatomy of maxillary first premolar by microscopic computed tomography in a Chinese adolescent subpopulation. Biomed. Res. Int..

[42] Maghfuri S., Keylani H., Chohan H., Dakkam S., Atiah A., Mashyakhy M. (2019). Evaluation of root canal morphology of maxillary first premolars by cone beam computed tomography in Saudi Arabian southern region subpopulation: An in vitro study. Int. J. Dent..

[43] Mashyakhy M., Gambarini G. (2019). Root and root canal morphology differences between genders: A comprehensive in-vivo CBCT study in a Saudi population. Acta Stomatol. Croat..

[44] Pan J.Y.Y., Parolia A., Chuah S.R., Bhatia S., Mutalik S., Pau A. (2019). Root canal morphology of permanent teeth in a Malaysian subpopulation using cone-beam computed tomography. BMC Oral Health.

[45] Rajakeerthi R., Nivedhitha M.S.B. (2019). Use of cone beam computed tomography to identify the morphology of maxillary and mandibular premolars in Chennai population. Braz. Dent. Sci..

[46] Saber S.E.D.M., Ahmed M.H.M., Obeid M., Ahmed H.M.A. (2019). Root and canal morphology of maxillary premolar teeth in an Egyptian subpopulation using two classification systems: A cone beam computed tomography study. Int. Endod. J..

[47] Asheghi B., Momtahan N., Sahebi S., Zangoie Booshehri M. (2020). Morphological evaluation of maxillary premolar canals in Iranian population: A cone-beam computed tomography study. J. Dent..

[48] Buchanan G.D., Gamieldien M.Y., Tredoux S., Vally Z.I. (2020). Root and canal configurations of maxillary premolars in a South African subpopulation using cone beam computed tomography and two classification systems. J. Oral Sci..

[49] Kfir A., Mostinsky O., Elyzur O., Hertzeanu M., Metzger Z., Pawar A.M. (2020). Root canal configuration and root wall thickness of first maxillary premolars in an Israeli population: A cone-beam computed tomography study. Sci. Rep..

[50] Nikkerdar N., Asnaashari M., Karimi A., Araghi S., Seifitabar S., Golshah A. (2020). Root and canal morphology of maxillary teeth in an Iranian subpopulation residing in western Iran using cone-beam computed tomography. Iran. Endod. J..

[51] Wu D., Hu D.Q., Xin B.C., Sun D.G., Ge Z.P., Su J.Y. (2020). Root canal morphology of maxillary and mandibular first premolars analyzed using cone-beam computed tomography in a Shandong Chinese population. Medicine.

[52] Al-Zubaidi S.M., Almansour M.I., Al Manour N.N., Alshammari A.S., Alshammari A.F., Altamimi Y.S., Madfa A.A. (2021). Assessment of root morphology and canal configuration of maxillary premolars in a Saudi subpopulation: A cone-beam computed tomographic study. BMC Oral Health.

[53] Dhaimy S., Dhoum S., Diouri M., Bedida L., Elmerini H., Benkiran I. (2021). Cone-beam computed tomography evaluation of the root morphology of the maxillary and mandibular premolars in a Moroccan subpopulation: Canal configurations and root curvatures (Part 2). Saudi Endod. J..

[54] Haider I., Sddique S.N., Tirmazi S.M., Moazzam M., Sana U., Rahseed M., Batool I. (2021). Evaluation of root canal morphology of maxillary first premolars by cone-beam computed tomography. Pak. J. Med. Health Sci..

[55] Malik S., Singla R., Gill G., Jain N., Kumar T., Arora S. (2021). Evaluation of root canal anatomy of maxillary premolars in a North Indian subpopulation using cone-beam computed tomography. Endodontology.

[56] Mashyakhy M. (2021). Anatomical evaluation of maxillary premolars in a Saudi population: An in vivo cone-beam computed tomography study. J. Contemp. Dent. Pract..

[57] Monardes H., Herrera K., Vargas J., Steinfort K., Zaror C., Abarca J. (2021). Root anatomy and canal configuration of maxillary premolars: A cone-beam computed tomography study. Int. J. Morphol..

[58] Yoza T., Serikawa M., Sugita T., Harada T., Usami A. (2021). Cone-beam computed tomography observation of maxillary first premolar canal shapes. Anat. Cell Biol..

[59] Aguilera J., Vallette M., Navarro P., Betancourt P. (2022). Root and root canal system morphology of maxillary first premolars in a Chilean subpopulation: A cone-beam computed tomography study. Int. J. Morphol..

[60] Alnaqbi H.S.Y., Gorduysus M.O., Al Shehadat S., Al Bayatti S.W., Mahmoud I. (2022). Evaluation of variations in root canal anatomy and morphology of permanent maxillary premolars among the Emirate population using CBCT. Open Dent. J..

[61] Gündüz H., Özlek E. (2022). Evaluation of root morphology and root canal configuration of mandibular and maxillary premolar teeth in Turkish subpopulation by using cone-beam computed tomography. East J. Med..

[62] Hanif F., Ahmed A., Javed M.Q., Khan Z.J., Ulfat H. (2022). Frequency of root canal configurations of maxillary premolars as assessed by cone-beam computed tomography scans in the Pakistani subpopulation. Saudi Endod. J..

[63] Iqbal A., Khattak O., Issrani R., Alonazi M.A., Ali A.H. (2022). Cone-beam computed tomography evaluation of root morphology of the premolars in Saudi Arabian subpopulation. Pesqui. Bras. Odontopediatria Clín. Integr..

[64] Medina-Guevara C.A., Oliva-Rodríguez R., Calvillo-Martínez D.H., Mariel-Cárdenas J., Muñoz-Ruiz A.I., Gutiérrez-Cantú F.J. (2022). Anatomical and morphological findings of the root canal of maxillary premolars and their prevalence: CBCT study in a Mexican population. Int. J. Morphol..

[65] Olczak K., Pawlicka H., Szymański W. (2022). Root form and canal anatomy of maxillary first premolars: A cone-beam computed tomography study. Odontology.

[66] Allawi S., Ayoubi H., Al-Tayyan M., Toutangy E., Tolibah Y.A. (2023). Evaluation of roots, root canal morphology, and bilateral symmetry of maxillary first molars in a Syrian subpopulation using cone-beam computed tomography. Clin. Exp. Dent. Res..

[67] Erkan E., Olcay K., Eyüboğlu T.F., Şener E., Gündoğar M. (2023). Assessment of the canal anatomy of the premolar teeth in a selected Turkish population: A cone-beam computed tomography study. BMC Oral Health.

[68] Khanna S., Jobanputra L., Mehta J., Parmar A., Panchal A., Mehta F. (2023). Revisiting premolars using cone-beam computed tomography analysis and classifying their roots and root canal morphology using newer classification. Cureus.

[69] Merhej M.J., El Hachem R., Sacre H., Salameh P., Ghosn N., Naaman A. (2023). The root canal morphology of premolars in a sample of the Lebanese population: Clinical considerations. Int. Arab J. Dent..

[70] Mirah M.A., Bafail A., Baik A., Abu Zaid B., Hakeem M., Ghabbani H. (2023). Root canal morphology of premolars in Saudis. Cureus.

[71] Retamoso-Palomino M., Mayta-Tovalino F., García-Rupaya C. (2023). A cone-beam computed tomography study of the root and root canal morphology of maxillary first premolars in young Peruvians. Saudi Endod. J..

[72] Shah S.A. (2023). Cone beam computed tomography evaluation of root canal morphology of maxillary premolars in North-West sub-population of Pakistan. Khyber Med. Univ. J..

[73] Akotiya B.R., Kassa S.C., Saha M.K., Saha S.G., Agarwal R.S., Balakrishnan S.P. (2024). Assessment of root morphology and canal configuration of maxillary first and second premolars in Central Indian population using CBCT and two classification systems—An observational study. Afr. J. Biol. Sci..

[74] Aljawhar A.M., Ibrahim N., Abdul Aziz A., Ahmed H.M.A., Azami N.H. (2024). Characterization of the root and canal anatomy of maxillary premolar teeth in an Iraqi subpopulation: A cone beam computed tomography study. Odontology.

[75] Mirza M.B. (2024). Evaluating the internal anatomy of maxillary premolars in an adult Saudi subpopulation using two classifications: A CBCT-based retrospective study. Med. Sci. Monit..

[76] Syed G.A., Pullishery F., Alhazmi K.A., Nazer M.I., Alkhamis A., Meer F.M., Halteet F.A., Sendiyoni A.M.I., Taher A.O.H. (2024). CBCT evaluation of root canal morphology of maxillary first premolar in Saudi subpopulation. J. Pharm. Bioallied Sci..

[77] Aljawhar A.M., Ibrahim N., Abdul Aziz A., Ahmed H.M.A., Azami N.H. (2025). Micro-computed tomographic evaluation of root and canal anatomy of maxillary first premolars in Iraqi sub-population. Sci. Rep..

[78] Almehrzi H., Khawajah S., Alharbi N.S., El Abed R., Jamal M. (2025). Evaluation of the root and canal morphology of maxillary and mandibular premolars in an Emirati sub-population. Int. Dent. Journal..

[79] Mustafa M., Karobari M.I., Al-Maqtari A.A.A., Abdulwahed A., Almokhatieb A.A., Almufleh L.S., Hashem Q., Alsakaker A., Alam M.K., Ahmed H.M.A. (2025). Investigating root and canal morphology of anterior and premolar teeth using CBCT with a novel coding classification system in Saudi subpopulation. Sci. Rep..

[80] Suresh S., Kalhoro F.A., Rani P., Memon M. (2024). Root canal morphology of premolars in population of Hyderabad, Pakistan: A cone beam computerised tomographic analysis. J. Coll. Physicians Surg. Pak..

[81] Yanqui-Gómez J.S., Dulanto-Vargas J.A., Carranza-Samanez K.M. (2024). Morphology of roots and canals of maxillary first premolars: A CBCT study in a Peruvian sample. Int. J. Dent..

[82] Jung Y.-H., Hwang J.-J., Lee J.-S., Cho B.-H. (2024). Analysis of root number and canal morphology of maxillary premolars using cone-beam computed tomography. Imaging Sci. Dent..

[83] Martins J.N.R., Tummala S., Nallapati S., Marques D.N.D.S., Silva E.S., Caramês J.M.M., Versiani M. (2025). Population-specific anatomical variations in premolar root canal systems: A cross-sectional cone-beam computed tomography study of Jamaican and Portuguese subpopulations. Dent. J..

[84] Acevedo-Tavie J., Méndez-Vera P., Ríos P., Rosas-Méndez C. (2024). Descripción de la morfología del sistema de canales radiculares en premolares maxilares mediante tomografía computarizada cone beam en una población chilena. Int. J. Odontostomatol..

[85] Hafiizh A.M.M., Yusuf S.N.M., Omar S.H., Mohammad N. (2025). Cone-beam computed tomography evaluation of canal morphology of maxillary premolars in Malaysian subpopulation using two canals classification. Endodontology.

[86] Watanabe S., Yabumoto S., Okiji T. (2024). Evaluation of root and root canal morphology in maxillary premolar teeth: A cone-beam computed tomography study using two classification systems in a Japanese population. J. Dent. Sci..

[87] Wolf T.G., Kozaczek C., Siegrist M., Betthäuser M., Paqué F., Briseño-Marroquín B. (2020). An ex vivo study of root canal system configuration and morphology of 115 maxillary first premolars. J. Endod..

[88] Alenezi M.A., Al-Nazhan S.A., Al-Omari M.A. (2022). Three-dimensional evaluation of root canal morphology of maxillary first premolars: Micro–computed tomographic study. Saudi Dent. J..

[89] Weine F.S., Healey H.J., Gerstein H., Evanson L. (1969). Canal configuration in the mesiobuccal root of the maxillary first molar and its endodontic significance. Oral Surg. Oral Med. Oral Pathol..

[90] Ahmed H.M.A., Versiani M.A., De-Deus G., Dummer P.M.H. (2017). A new system for classifying root and root canal morphology. Int. Endod. J..

[91] Liberati A., Altman D.G., Tetzlaff J., Mulrow C., Gøtzsche P.C., Ioannidis J.P., Clarke M., Devereaux P.J., Kleijnen J., Moher D. (2009). The PRISMA statement for reporting systematic reviews and meta-analyses of studies that evaluate health care interventions: Explanation and elaboration. PLoS Med..

[92] Higgins J.P., Thompson S.G. (2002). Quantifying heterogeneity in a meta-analysis. Stat. Med..

[93] Henry B.M., Tomaszewski K.A., Ramakrishnan P.K., Roy J., Vikse J., Loukas M., Tubbs R.S., Walocha J.A. (2017). Development of the anatomical quality assessment (AQUA) tool for the quality assessment of anatomical studies included in meta-analyses and systematic reviews. Clin. Anat..

[94] Page M.J., McKenzie J.E., Bossuyt P.M., Boutron I., Hoffmann T.C., Mulrow C.D., Shamseer L., Tetzlaff M.J., Akl A.E., Brennan E.S. (2021). The PRISMA 2020 statement: An updated guideline for reporting systematic reviews. BMJ.

[95] Plotino G., Grande N.M., Pecci R., Bedini R., Pameijer C.H., Somma F. (2006). Three-dimensional imaging using microcomputed tomography for studying tooth macromorphology. J. Am. Dent. Assoc..

[96] Limoeiro A.G., Braitt A.H., Machado A.S., Bueno C.E., Fontana C.E., Freire L.G., Lopes R.T., De Martin A.S. (2021). Micro-computed Tomography Evaluation of Filling Material Removal by Three Reciprocating Systems with Different Thermal Treatments. Giorn. Ital. Endod..

[97] Ahmed H.M.A., Keleş A., Wolf T.G., Nagendrababu V., Duncan H.F., Peters O.A., Dummer P.M.H. (2024). Controversial Terminology in Root and Canal Anatomy: A Comprehensive Review. Eur. Endod. J..

[98] Spagnuolo G., Ametrano G., D’Antò V., Formisano A., Simeone M., Riccitiello F., Amato M., Rengo S. (2012). Microcomputed Tomography Analysis of Mesiobuccal Orifices and Major Apical Foramen in First Maxillary Molars. Open Dent. J..

