# Supplementary material for: Maxillary First Premolars’ Internal Morphology: A Systematic Review and Meta-Analysis
Source: Dent J (Basel). 2025 Nov 3;13(11):510. doi: 10.3390/dj13110510 (PMC12651017; doi:10.3390/dj13110510)
Supplement: Supplementary file 1 [file dentistry-13-00510-s001.zip › S3. Supplementary Materials_AQUA tool evaluation.pdf]

Supplement - AQUA Tool evaluation

Study	Risk of bias				
	**Target and subject attributed**	**Design of the study**	**Methodology description**	**Descriptive anatomy**	**Reporting of outcomes**
Pineda & Kuttler, 1972	Low	Low	High	Low	Low
Carns & Skidmore, 1973	Low	Low	High	Low	Low
Green D., 1973	Unclear	Low	High	Low	Low
Vertucci & Gegauff, 1979	Low	Low	High	Low	Low
Caliskan et al., 1995	Low	Low	Low	Low	Low
Kartal et al., 1998	Low	Low	Low	Low	Low
Sert & Bayirli, 2004	Low	Low	Low	Low	Low
Atieh, M. A., 2008	Low	Low	High	Unclear	Low
Awawdeh et al., 2008	Low	Low	Low	Low	Low
Peiris et al., 2008	Low	Low	Low	Low	Low
Weng et al., 2009	Low	Low	Low	Low	Low
Ng'Ang'A et al., 2010	Low	Low	High	Low	Low
Neelakantan et al., 2011	Low	Low	High	Low	Low
Özcan et al., 2012	Low	Low	High	Low	Low
Tian et al., 2012	Low	Low	Low	Low	Low
Ok et al., 2014	Low	Low	Low	Low	Low
Abella et al., 2015	Low	Low	Low	Low	Low
Bulut et al., 2015	Low	Low	Low	Low	Low

Felyspremila et al., 2015	Low	Low	Low	Low	Low
Gupta et al., 2015	Low	Low	Low	Low	Low
Celikten et al., 2015	Low	Low	Low	Low	Low
Bürklein et al., 2017	Low	Low	Low	Low	Low
Martins et al., 2017	Low	Low	Low	Low	Low
Shi et al., 2017	Low	Low	Low	Low	Low
Alqedairi et al., 2018	Low	Low	Low	Low	Low
Dinakar et al., 2018	Low	Low	High	Low	Low
Li et al., 2018	Low	Low	Low	Low	Low
Martins et al., 2018	Low	Low	Low	Low	Low
Martins et al., 2018	Low	Low	Low	Low	Low
Martins et al., 2018	Low	Low	Low	Low	Low
Nazeer et al., 2018	Low	Low	Low	Low	Low
Senan et al., 2018	Low	Low	Low	Low	Low
de Lima et al., 2019	Low	Low	Low	Low	Low
Liu et al., 2019	Low	Low	Low	Low	Low
Maghfuri et al., 2019	Low	Low	Low	Low	Low
Mashyakhy & Gambarini., 2019	Low	Low	Low	Low	Low
Pan et al., 2019	Low	Low	Low	Low	Low
Rajakeerthi & Nivedhitha, 2019	Low	Lowv	Low	Low	Low
Saber et al., 2019	Low	Low	Low	Low	Low

Asheghi et al., 2020	Low	Low	Low	Low	Low
Buchanan et al., 2020	Low	Low	Low	Low	Low
Kfir et al., 2020	Low	Low	Low	Low	Low
Nikkerdar et al., 2020	Low	Low	Low	Low	Low
Wolf et al., 2020	Low	Low	Low	Low	Low
Wu et al., 2020	Low	Low	Low	Low	Low
Agholor & Sede., 2021	Low	Low	Low	Low	Low
Al-Zubaidi et al., 2021	Low	Low	Low	Low	Low
Dhaimy et al., 2021	Low	Low	Low	Low	Low
Haider et al., 2021	Low	Low	Low	Unclear	Low
Malik et al., 2021	High	Low	Low	Low	Low
Mashyakhy, M., 2021	Low	Low	Low	Low	Low
Monardes et al., 2021	Low	Low	Low	Low	Low
Qiao et al., 2021	High	Low	Low	Low	Low
Yoza et al., 2021	Low	Low	Low	Low	Low
Aguilera et al., 2022	Low	Low	Low	Low	Low
Alenezi et al., 2022	Low	Low	Low	Low	Low
Alnaqbi et al., 2022	Low	Low	Low	Low	Low
Faraj et al., 2022	Low	Low	Low	Low	Low
Gündüz & Özlek., 2022	Low	Low	Low	Low	Low
Hanif et al., 2022	Low	Low	Low	Low	Low
Iqbal et al., 2022	Low	Low	Low	Low	Low
Khattak et al., 2022	Low	Low	Low	Low	Low

Medina - Guevara et al., 2022	Low	Low	High	Unclear	Low
Kartik et al., 2022	Low	Low	High	Unclear	Low
Olzcak et al., 2022	Low	Low	Low	Low	Low
Peiris et al., 2022	Low	Low	High	Low	Low
Allawi et al., 2023	Low	Low	Low	Low	Low
Erkan et al., 2023	Low	Low	Low	Low	Low
Khanna et al., 2023	Low	Low	High	Low	Low
Merhej et al., 2023 (preprint)	Low	Low	Low	Low	Low
Mirah et al., 2023	Low	Low	Low	Low	Low
Retamoso-Palomino et al., 2023	Low	Low	Low	Low	Low
Shah et al., 2023	Low	Low	Low	Low	Low
Akotiya et al., 2024	Low	Low	Low	Low	Low
Aljawhar et al., 2024	Low	Low	Low	Low	Low
Mirza et al., 2024	Low	Low	Low	Low	Low
Syed et al., 2024	Low	Low	High	Low	Low
Aljawhar et al., 2025	Low	Low	Low	Low	Low
Almehrzi et al., 2025	Low	Low	Low	Low	Low
Mustafa et al., 2025	Low	Low	Low	Low	Low
Suresh et al., 2024	Low	Low	Low	Low	Low
Yanqui-Gomez et al., 2024	Low	Low	Low	Low	Low

Jung et al., 2024	Low	Low	Low	Low	Low
Martins et al,. 2025	Low	Low	Low	Low	Low
Acevedo-Tavie et al., 2024	Low	Low	Low	Low	Low
Hafiizh et al., 2025	Low	Low	Low	Low	Low
Watanabe et al., 2025	Low	Low	Low	Low	Low
