# Supplementary material for: Maxillary First Premolars’ Internal Morphology: A Systematic Review and Meta-Analysis
Source: Dent J (Basel). 2025 Nov 3;13(11):510. doi: 10.3390/dj13110510 (PMC12651017; doi:10.3390/dj13110510)
Supplement: Supplementary file 1 [file dentistry-13-00510-s001.zip › S4. Table 1. Characteristics of the included studies of Mx1Ps categorized by authors, study type, teeth investigated, methodology, and country.pdf]

Table 1. Characteristics of the included studies of Mx1Ps categorized by authors, study type, teeth investigated, methodology, language and country of origin

ID	Author	Type of study	Teeth	Method of study	Language	Country
1	Pineda & Kuttler [4]	*In vitro*	Permanent	Rx	English	Mexico
2	Carns & Skidmore [24]	*In vitro*	Maxillary first premolars	Plastic casts/Hydrogen peroxide	English	USA
3	Green [9]	*In vitro*	Permanent	Grinding/examination under X3 magnification/red dye	English	USA
4	Vertucci & Gegauff [10]	*In vitro*	Maxillary first premolars	Clearing/dye	English	USA
5	Calişkan et al. [11]	*In vitro*	Permanent	Clearing/stereomicroscope X12 magnification	English	Turkey
6	Kartal et al. [12]	In vitro	Maxillary premolars	Clearing/dye/light microscope	English	Turkey
7	Sert & Bayirli [13]	*In vitro*	Permanent	Clearing/Ink	English	Turkey
8	Atieh [5]	*In vitro*	Maxillary first premolars	Clearing/Rx/Cross-Sections	English	Saudi Arabia
9	Awawdeh et al. [14]	*In vitro*	Maxillary first premolars	Clearing/Stereomicroscope X3 magnification	English	Jordan
10	Peiris [15]	*In vitro*	Permanent	Clearing/X10 magnification	English	Japan/Sri Lanka
11	Weng et al. [16]	*In vitro*	Maxillary permanent	Clearing/ink	English	China (subpopulation)
12	Ng'Ang'A et al. [17]	*In vitro*	Maxillary first premolars	Clearing/ink/microscope X10 and X40	English	Kenya
13	Neelakantan et al. [18]	*In vitro*	Maxillary premolars	Clearing/X3 microscope	English	India
14	Özcan et al. [2]	*In vitro*	Maxillary first premolars	Rx/Cross-Sections	English	Turkey

15	Tian et al. [25]	Retrospective	Maxillary first premolars	CBCT	English	China (subpopulation)
16	Ok et al. [26]	Retrospective	Maxillary and mandibular premolars	CBCT	English	Turkey
17	Abella et al. [27]	Retrospective	Maxillary premolars	CBCT	English	Spain
18	Bulut et al. [28]	Retrospective	Maxillary and mandibular premolars	CBCT	English	Turkey
19	Felsypremila et al. [29]	Retrospective	Maxillary and mandibular posterior	CBCT	English	India (Subpopulation)
20	Gupta et al. [19]	*In vitro*	Maxillary first premolars	Clearing/Stereomicroscope	English	North India
21	Celikten et al. [30]	Retrospective, cross-sectional	Maxillary and mandibular premolars	CBCT	English	Turkey (Cypriot subpopulation)
22	Bürklein et al. [31]	Retrospective	Maxillary and mandibular premolars	CBCT	English	Germany
23	Martins et al. [32]	Retrospective	Permanent	CBCT	English	Portugal
24	Shi et al. [33]	Retrospective	Maxillary premolars	CBCT	English	China
25	Alqedairi et al. [34]	Retrospective, cross-sectional	Maxillary premolars	CBCT	English	Saudi Arabia
26	Dinakar et al. [20]	*In vitro*	Maxillary first premolars	Clearing/sterepmicroscope	English	South India

27	Li et al. [35]	Retrospective	Maxillary premolars	CBCT	English	China
28	Martins et al. [36]	Retrospective	Permanent teeth	CBCT	English	China/Portugal
29	Martins et al. [37]	Retrospective	Permanent teeth	CBCT	English	Portugal
30	Martins et al. [38]	Retrospective	Permanent teeth	CBCT	English	Poland
31	Nazeer et al. [39]	Retrospective	Maxillary premolars	CBCT	English	Pakistan
32	Senan et al. [21]	*In vitro*	Maxillary first premolars	Clearing/ink	English	Yemen
33	De Lima et al. [40]	Retrospective, cross-sectional	Maxillary premolars	CBCT	English	Brazil
34	Liu et al. [41]	Retrospective	Maxillary first premolars	CBCT	English	China
35	Maghfuri et al. [42]	Retrospective	Maxillary first premolars	CBCT	English	Saudi Arabia
36	Mashyakhy & Gambarini [43]	Retrospective	Permanent teeth	CBCT	English	Saudi Arabia
37	Pan et al. [44]	Retrospective	Permanent teeth	CBCT	English	Malaysia
38	Rajakeerthi & Nivedhita [45]	Retrospective	Maxillary and mandibular premolars	CBCT	English	India (Subpopulation)
39	Saber et al. [46]	Retrospective	Maxillary premolars	CBCT	English	Egypt
40	Asheghi et al. [47]	Retrospective, cross-sectional	Maxillary premolars	CBCT	English	Iran
41	Buchanan et al. [48]	Retrospective, cross-sectional	Maxillary premolars	CBCT	English	South Africa
42	Kfir et al. [49]	Retrospective	Maxillary premolars	CBCT	English	Israel

43	Nikkerdar et al. [50]	Retrospective	Maxillary teeth	CBCT	English	Iran
44	Wolf et al. [77]	Retrospective	Maxillary first premolars	Micro-CT	English	Switzerland/Germany
45	Wu et al. [51]	Retrospective	Maxillary and mandibular first premolars	CBCT	English	China (Subpopulation)
46	Agholor & Sede [6]	*In vivo*	Maxillary premolars	Rx	English	Nigeria
47	Al-Zubaidi et al. [52]	Retrospective	Maxillary premolars	CBCT	English	Saudi Arabia
48	Dhaimy et al. [53]	Retrospective	Maxillary and mandibular premolars	CBCT	English	Morocco
49	Haider et al. [54]	Retrospective	Maxillary first premolars	CBCT	English	Pakistan
50	Malik et al. [55]	Retrospective	Maxillary premolars	CBCT	English	Indianorth)
51	Mashyakhy [56]	Retrospective, cross-sectional	Permanent teeth	CBCT	English	Saudi Arabia
52	Monardes et al. [57]	Retrospective	Maxillary premolars	CBCT	English	Chile
53	Qiao et al. [7]	*In vitro*	Maxillary posterior teeth	Clearing/Rx	English	China (Subpopulation)
54	Yoza et al. [58]	Retrospective	Maxillary first premolars	CBCT	English	Japan
55	Aguilera et al. [59]	Retrospective	Maxillary first premolars	CBCT	English	Chile
56	Alenezi et al. [78]	*In vitro*	Maxillary first premolars	Micro-CT	English	Saudi Arabia

57	Alnaqbi et al. [60]	Retrospective	Maxillary premolars	CBCT	English	United Arabic Emirates
58	Faraj et al. [8]	*In vitro*	Maxillary first premolars	Rx/Cross-sectioning	English	Iraq
59	Gündüz & Özlek [61]	Retrospective	Maxillary and mandibular premolars	CBCT	English	Turkey
60	Hanif et al. [62]	Retrospective	Maxillary premolars	CBCT	English	Pakistan
61	Iqbal et al. [63]	Retrospective	Maxillary and mandibular premolars	CBCT	English	Saudi Arabia
62	Khattak et al. [22]	Retrospective	Maxillary first premolars	Clearing/Ink/Cross-sections	English	Pakistan (Subpopulation)
63	Medina-Guevara et al. [64]	Retrospective	Maxillary premolars	CBCT	English	Mexico
64	Olczak et al. [65]	Retrospective	Maxillary first premolars	CBCT	English	Poland
65	Peiris et al. [23]	*In vitro*	Permanent teeth	Clearing/X10 magnification	English	Sri Lanka, Japan
66	Allawi et al. [66]	*In vitro*	Maxillary first premolars	CBCT	English	Syria
67	Erkan et al. [67]	Retrospective	Maxillary and mandibular premolars	CBCT	English	Turkey
68	Khanna et al. [68]	Retrospective	Maxillary and mandibular premolars	CBCT	English	India (Subpopulation)

69	Merhej et al. [69]	Retrospective	Maxillary and mandibular premolars	CBCT	English	Lebanon
70	Mirah et al. [70]	Retrospective	Maxillary and mandibular premolars	CBCT	English	Saudi Arabia
71	Retamoso-Palomino et al. [71]	Retrospective	Maxillary first premolars	CBCT	English	Peru
72	Shah [72]	Retrospective	Maxillary premolars	CBCT	English	Pakistan (Subpopulation)
73	Akotiya et al. [73]	Retrospective	Maxillary premolars	CBCT	English	India
74	Aljawhar et al. [74]	Retrospective	Maxillary premolars	CBCT	English	Irak
75	Mirza et al. [75]	Retrospective	Maxillary premolars	CBCT	English	Saudi Arabia (Subpopulation
76	Syed et al. [76]	Retrospective	Maxillary first premolars	CBCT	English	Saudi Arabia (Subpopulation)
77	Aljawhar et al. [77]	Retrospective	Maxillary first premolars	CBCT	English	Irak
78	Almehrzi et al. [78]	Retrospective	Maxillary first premolars	CBCT	English	United Arab Emirates
79	Mustafa et al. [79]	Retrospective	Anterior and premolar teeth	CBCT	English	Saudi Arabia
80	Suresh et al. [80]	Retrospective	Maxillary premolars	CBCT	English	Pakistan
81	Yanqui-Gómez et al. [81]	Retrospective	Maxillary first premolars	CBCT	English	Peru

82	Jung et al. [82]	Retrospective	Maxillary premolars	CBCT	English	Korea
83	Martins et al. [83]	Retrospective	Maxillary and mandibular premolars	CBCT	English	Jamaica/Portugal
84	Acevedo-Tavie et al. [84]	Retrospective	Maxillary premolars	CBCT	Spanish	Chile
85	Hafiizh et al. [85]	Retrospective	Maxillary premolars	CBCT	English	Malaysia
86	Watanabe et al. [86]	Retrospective	Maxillary premolars	CBCT	English	Japan
