# Supplementary material for: Maxillary First Premolars’ Internal Morphology: A Systematic Review and Meta-Analysis
Source: Dent J (Basel). 2025 Nov 3;13(11):510. doi: 10.3390/dj13110510 (PMC12651017; doi:10.3390/dj13110510)
Supplement: Supplementary file 1 [file dentistry-13-00510-s001.zip › S5. Table 2. Morphologic investigations of the RCC of Mx1Ps.pdf]

Table 2. Morphologic investigations of the root canal configurations (RCCs) of Mx1Ps. The RCCs are depicted according to the classifications of Weine et al. (We)[79], Vertucci (Ve)[1] and Briseño Marroquín et al. (Br)[3] (PP: Country three-digit code of population investigated; Met: Research methodology employed; -: no classification given/possible; CHN*: Chinese subpopulation; IND*: Indian subpopulation Cl: clearing method; Rx: radiographic method; R: Resin; GR: grinding method; SC: staining and clearing; Mic: under microscopic observation; CR: cross-sectional method; CBCT: Cone-beam computed tomography; m-CT: micro CT; C: Plastic cast method F: Female; M: Male; 14: Right maxillary first premolar; 24: Left maxillary first premolar.

Report	PP	n	Met	Root canal configuration frequency (%)									Number if roots (%)		
	RCC		Ve	I	II	III	IV	V	VI	VII	VIII	*	1	2	3
			We	I	II	-	III	IV	-	-	-	*			
			Br	1-1-1/1	2-2-1/1	1-2-1/1	2-2-2/2	1-1-2/2	2-1-2/2	1-2-1/2	1-1-3/3	*			
Pineda & Kuttler [4]	MEX	259	Rx	26.2	23.9	0	41.7	7.7	0	0	0.5	0	-	-	-
Carns & Skidmore, [24]	USA	100	C	9	0	13.0	57.0	15.0	0	0	6.0	0	-	-	-
Green [9]	USA	50	GR	8.0	26.0	0	66.0	0	0	0	0	0	-	-	-
Vertucci, [10]	USA	400	SC	8.0	18.0	0	62.0	7.0	0	0	5.0	0	39.5	56.5	4.0
Caliskan et al. [11]	TUR	100	SC	3.9	5.9	0	78.4	5.9	5.9	0	0	0	-	-	-
Kartal et al. [12]	TUR	300	SC	8.7	1.0	0	71.3	12.0	2.7	0.3	1.3	0.3	37.3	61.3	1.3
Sert & Bayirli [13]	TUR	100	SC; Mic M	9.0	13.0	6.0	60.0	5.0	0	2.0	2.0	3.0	-	-	-
		100	SC; Mic F	12.0	12.0	5.0	63.0	2	2	0	4	0	-	-	-
Atieh, [5]	SAU	246	CR; Rx	8.9	26.8	0	63.0	0	0	0	1.2	0	17.9	80.9	1.2
Awawdeh et al. [14]	JOR	600	SC	3.3	10.2	0.3	79.7	2	2.3	0	1.5	0	30.8	68.4	0.8
Peiris, [15]	LKA	153	SC	1.3	16.3.	2.0	64.0	5.9	5.9	0.7	0	3.9	68.0	32.0	-

	JPN	81		4.9	29.6	2.5	45.7	2.5	8.6	0	0	6.2	87.7	11.1	1.2
Weng et al. [16]	CHN*	95	SC	6.3	22.1	3.2	64.2	3.2	1.0	0	0	0	12.7	87.3	0
Ng’Ang’A. et al. [17]	KEN	155	SC	1.9	2.6	1.9	71.6	12.6	0.7	1.9	6.5	0	6.5	87.1	6.5
		77	M	2.6	2.6	0	68.8	14.3	0	0	11.7	0	5.2	83.1	11.7
		78	F	1.3	2.6	3.8	74.4	11.5	1.3	3.8	1.3	0	7.7	91.0	1.3
Neelakantan et al. [18]	IND	350	SC total	-	-	-	-	-	-	-	-	-	11.7	86.0	2.2
		41	Single root	21.9	34.1	7.5	26.8	9.7	0	0	0	0	-	-	-
		294	Two separate roots. B	33.0	14.0	19.7	11.9	20.4	0	0	0	1.02	-	-	-
			Two separate roots, P	84.4	10.8	0	0	0	4.8	0	0	0	-	-	-
		7	Two fused roots, B	28.6	14.3	0	14.3	14.3	0	0	0	28.5	-	-	-
			Two fused roots, P	57.2	0	0	28.6	14.2	0	0	0	0	-	-	-
		6	Three roots, B	16.6	16.6	16.6	16.6	33.6	0	0	0	0	-	-	-
			Three roots, MP	16.6	0	0	0	83.4	0	0	0	0	-	-	-
			Three roots, DP	16.6	0	16.6	0	66.8	0	0	0	0	-	-	-

		2	Three roots, MB	50	0	50.0	0	0	0	0	0	0	-	-	-
			Three roots DB	50	0	0	0	50.0	0	0	0	0	-	-	-
			Three roots P	50	0	50.0	0	0	0	0	0	0	-	-	-
Özcan et al. [2]	TUR	653	SC/Rx/CR	7.8	22.2	0	68.5	0	0	0	1.5	0	45.2	53.7	1.1
Tian et al. [25]	CHN*	300	CBCT total	14	23	4	51	3	2	1	1	0	66.0	33.0	1.0
		198	Single root	22	35	7	29	4	3	1	0	0	-	-	-
		100	Two roots	0	0	0	96	2	0	0	0	0	-	-	-
		2	Three roots	0	0	0	0	0	0	0	100.0	0	-	-	-
Ok et al. [26]	TUR	200	CBCT total	9.57	6.46	1.38	76.94	4.57	0.7	0	1.01	0	-	-	-
			14 M	5.3	6.2	0.8	80.4	4.5	0.3	0	2.5	0	-	-	-
			14 F	11.5	7.5	1.7	75	4.3	0	0	0	0	-	-	-
			24 M	7.7	5.6	1.2	78.7	5.3	0	0	1.5	0	-	-	-
			24 F	14	6.5	1.8	73.5	4.2	0	0	0	0	-	-	-
Abella et al. [27]	SPA	430	CBCT total	25.1	10.2	4.4	52.8	1.9	1.6	1.4	2.6	0	46.0	51.4	2.6

		198	Single root	54.5	22.2	9.6	6.1	3.0	2.0	2.5	0	0	-	-	-
		221	Two roots	0	0	0	97.3	0.9	1.4	0.5	0	0	-	-	-
		11	Three roots	0	0	0	0	0	0	0	100.0	0	-	-	-
Bulut et al. [28]	TUR	511	CBCT	62.6	34.1	0.8	1.9	0.6	0	0	0	0.1	28.2	70.8	1
Felsypremila et al., [29]	IND	418	CBCT	15.9	26.1	0	58	0	0	0	0	0	-	-	-
Gupta et al. [19]	IND*	250	SC	23.2	14.8	13.6	33.2	6.8	2	4	0.4	2	53.6	46	0.4
Celikten et al. [30]	TUR	437	CBCT total	4.5	16.2	0.4	76.8	0.6	0	0	0.9	0	53.7	44.8	0.9
Bürklein et al. [31]	GER	644	CBCT total	3.9	6.5	0	68.5	7.9	12.3	0.2	2	0	36.4	62.4	1.2
		322	14 Total	3.4	7.1	0	69.3	8.7	9.3	0.3	1.9	0	35.1	63.7	1.2
		322	24 Total	4.3	5.9	0	67.7	7.1	12.7	0	2	0	37.6	61.2	1.2
		302	M Total	1.4	3.3	0	76.2	7.3	10	0	2.0	0	26.5	72.2	1.4
		342	F Total	5.9	9.4	0	61.8	8.2	12.6	0.3	2	0	45.1	53.9	1.2
Martins et al. [32]	PRT	690	CBCT	3.2	17.3	0.3	68	0.9	4.8	0	0.7	4.8	48.7	49.1	2.2
Shi et al. [33]	CHN	521	CBCT	4.0	28.8	0	52.0	1.34	9.0	0.2	1.9	2.7	60.80	37.8	1.34

		317	Single root	6.6	47.3	0	24.6	2.2	14.8	0.3	0	4.1	-	-	-
		197	Two roots	0	0	0	98.0	0	0	0	0.03	0	-	-	-
		7	Three roots	0	0	0	0	0	0	0	85.7	14.3	-	-	-
Alqedairi et al. [34]	SAU	334	CBCT	10.8	8.4	1.8	70.6	3.9	2.1	0.3	2.1	0	23.7	75.1	1.2
		172	M	12.2	6.4	0.6	72.1	2.3	2.3	0.6	3.5	0	20.9	76.8	2.3
		162	F	9.3	10.5	3.1	69.1	5.6	1.8	0	0.6	0	26.5	73.5	0
Dinakar et al. [20]	IND	225	SC	9.8	13.4	0.5	66.5	0.5	6.5	0	2.3	0	52.9	44.9	2.2
Li et al. [35]	CHN	1387	CBCT total	11.8	14.7	16.1	42.7	12.1	0.6	0.7	0.7	0.6	69.7	29.8	0.5
		967	Single root	16.9	21.1	23.1	19.0	17.2	0.9	1.0	0	0.8	-	-	-
		413	Two roots	0	0	0	98.8	0.5	0	0	0.7	0	-	-	-
		7	Three roots	0	0	0	0	0	0	0	100.0	0	-	-	-
Martins et al. [36]	CHN	238	CBCT	12.2	22.7	4.2	55.0	5.9	0	0	0	0	83.2	16.8	0
	PRT	714		3.4	17.1	0.3	68.2	1.0	4.6	0	0.7	4.7	48.7	49.2	2.1
Martins et al. [37]	PRT	269	CBCT M	0.7	10.4	0.4	74.0	1.1	4.1	0	1.9	7.4	34.6	62.1	3.3
		445	CBCT F	4.9	21.1	0.2	64.7	0.9	4.9	0	3.2	0.98	57.3	41.3	1.3
Martins et al. [38]	PRT	10	CBCT ≤20	0	0	0	90.0	0	0	0	0	10.0	-	-	-
		256	21-40	4.3	20.7	0	63.3	2.3	5.1	0	0.4	3.9	-		-
		298	41-60	3.0	13.4	0.7	72.5	0.3	4.7	0	1.4	4.0	-	-	-

		150	≥61	2.6	19.3	0	66.7	0	4.0	0	0	7.4	-	-	-
Nazeer et al. [39]	PAK	114	CBCT	68.0	12.9	7.5	0	3.74	3.2	0	0.5	4.27	31.5	68.5	0
Senan et al. [21]	YEM	250	SC	13.2	4.4	8.0	55.6	5.6	1.6	3.6	0.8	7.2	54.8	44.4	0.8
De Lima et al. [40]	BRA	496	CBCT total	6.5	7.7	0.6	82.2	0.8	0.6	0	1.6	0	18.2	80.2	1.6
		90	Single root	35.6	42.2	3.3	16.7	0	2.2	0	0	0	-	-	-
		398	Two roots	0	0	0	98.7	1.05	0.25	0	0	0	-	-	-
		8	Three roots	0	0	0	0	0	0	0	100	0	-	-	-
Liu et al. [41]	CHN	324	CBCT Total	25.0	8.3	2.8	52.8	5.6	2.8	1.5	1.2	0	72.2	26.5	1.2
Maghfuri et al. [42]	SAU	100	CBCT	0	7	0	75	13	2	0	3	0	36.0	61.0	3.0
Mashyakhy & Gambarini [43]	SAU	175	CBCT M	5.1	10.3	4.0	68.0	8.6	0	0	0	4.0	36.0	61.7	2.3
		176	CBCT F	2.3	3.4	11.4	59.7	21.0	0.6	0	0	1.7	45.5	50.1	1.1
Pan et al. [44]	MAL	304	CBCT	17.0	23.8	15.5	22.3	5.3	13.6	2.4	0	0	67.8	31.9	0.3
Rajakeerthi & Nivedhitha [45]	IND	200	CBCT Total	12.5	10.8	12.2	30.4	18.9	3.8	5.7	5.7	0	36.3	56.7	7.0
			14	10.0	9.0	6.0	42.0	23.0	2.0	2.0	6.0	0	43.0	51.0	6.0
			24	15.0	13.0	18.0	20.0	14.0	6.0	9.0	5.0	0	29.0	64.0	7.0
			M	13.2	12.3	10.5	34.2	15.8	5.3	4.4	4.4	0	34.2	62.3	3.5
			F	11.6	9.3	14.0	26.7	22.1	2.3	7.0	7.0	0	38.4	51.2	10.5

Saber et al. [46]	EGY	358	CBCT	1.1	15.6	1.4	73.2	1.4	3.9	1.7	1.4	0.3	45.8	53.1	1.1
Asheghi et al. [47]	IRN	462	CBCT total	8.9	15.1	0.9	71.6	1.3	0.2	0	2.0	0	50.2	48.1	1.7
		184	M	-	-	-	-	-	-	-	-	-	40.2	57.6	2.2
		278	F	-	-	-	-	-	-	-	-	-	56.8	41.7	1.4
		231	14	-	-	-	-	-	-	-	-	-	48.5	49.8	1.7
		231	24	-	-	-	-	-	-	-	-	-	52.0	46.3	1.7
		232	Single root	17.7	30.2	1.7	47.4	2.6	0.4	0	0	0	0	-	-
		222	Two roots	0	0	0	99.6	0	0	0	0.45	0	0	-	-
		8	Three roots	0	0	0	0	0	0	0	100.0	0	0	-	-
Buchanan et al. [48]	ZAF	316	CBCT total	8.9	7.3	4.7	71.8	2.2	2.2	0	2.8	0	44.0	54.1	1.9
		142	M	-	-	-	-	-	-	-	-	-	38.7	57.7	3.5
		174	F	-	-	-	-	-	-	-	-	-	48.3	51.1	1.9
		139	Single root	20.1	16.5	10.8	43.9	3.6	5.0	0	0	0	-	-	-
		171	Two roots	0	0	0	97.0	1.2	0	0	0	0	-	-	-
		6	Three roots	0	0	0	0	0	0	0	100.0	0	-	-	-
Kfir et al. [49]	ISR	400	CBCT	2	17	0.5	74	0.5	6	0	0	0	36.0	61.0	3.0
Nikkerdar et al. [50]	IRN	125	CBCT	18.4	2.4	20.0	9.6	40.0	12.0	0	0	0	75.2	24.8	0

Wolf et al. [87]	CHE	115	m-CT	0	2.6	0	47.8	3.5	4.3	2.6	3.5	0	26.1	70.0	3.5
Wu et al. [51]	CHI	1268	CBCT	10.4	24.2	0.39	58.6	4.9	0.8	0	0.3	0.24	67.4	32.0	0.55
Agholor & Sede [6]	NGA	24	Rx	12.5	20.8	0	66.7	0	0	0	0	0	33.3	66.7	0
Al-Zubaidi et al. [52]	SAU	500	CBCT total	5.2	32.8	0.6	57.8	2.0	0	0	1.6	0	39.8	58.6	1.6
			M	-	-	-	-	-	-	-	-	-	29.3	67.2	3.4
			F	-	-	-	-	-	-	-	-	-	48.9	51.1	0
			14	-	-	-	-	-	-	-	-	-	37.6	60.8	1.6
			24	-	-	-	-	-	-	-	-	-	42.0	56.4	1.6
Dhaimy et al. [53]	MAR	180	CBCT total	-	-	-	-	-	-	-	-	-	-	-	-
		69	Single root	4.4	7.2	20.4	15.9	7.2	17.4	27.5	0	0	-	-	-
		111	Two roots, B	46.8	0.9	0.9	4.5	45.0	0	1.8	0	0	-	-	-
		111	Two roots, P	46.8	3.6	2.7	39.6	0	0.9	2.7	0	0	-	-	-
Haider et al. [54]	PAK	200	CBCT	72.0	4.0	3.0	5.0	13.0	2.0	1.0	0	0	21	75	4
Malik et al. [55]	IND*	52	CBCT total	23.1	7.7	5.8	34.6	17.3	3.8	7.7	0	0	42.3	57.7	0
		22	Single root	54.5	18.2	13.6	4.6	9.1	0	0	0	0	-	-	-
		30	Two roots	0	0	0	56.7	23.3	6.7	13.3	0	0	-	-	-
	SAU	351	CBCT	3.7	6.8	7.7	63.8	14.8	0.3	0	0	0	40.7	57.5	1.7

Mashyakhy et al. [56]			total												
		177	14	4.5	6.2	7.9	63.3	14.7	0.6	0	0	2.8	41.8	56.5	1.7
		174	24	2.9	7.5	7.5	64.4	14.9	0	0	0	2.9	39.7	58.6	1.7
Monardes et al. [57]	CHL	306	CBCT	11.1	18	4.2	51.3	4.5	0	0	5.6	5.2	41.3	51.3	5.6
Qiao et al. [7]	CHN*	50	Cl/Rx	12.0	26.0	6.0	44.0	6.0	2.0	2.0	0.0	0	-	-	-
Yoza et al. [58]	JAP	125	CBCT	25.6	18.4	3.2	37.6	0.8	0	4	0.8	9.6	-	-	-
Aguilera et al. [59]	CHL	121	CBCT total	31.4	16.5	9.9	41.3	0.8	0	0	0	0	71.1	28.9	0
		26	M 14	23.1	19.2	7.7	50.0	0.0	0	0	0	0	-	-	-
		25	M 24	24.0	20.0	8.0	48.0	0.0	0	0	0	0	-	-	-
		35	F 14	40.0	11.4	11.4	34.3	2.9	0	0	0	0	-	-	-
		35	F 24	34.4	17.1	11.4	37.1	0.0	0	0	0	0	-	-	-
Alenezi et al. [88]	SAU	100	m-CT Total	9.0	11.0	5.0	53.0	5.0	11.0	1.0	4.0	1.0	57.0	41.0	2.0
		57	Single root	9	11	5	14	5	11	1	0	1	-	-	-
		41	Two roots	0	0	0	39	0	0	0	2	0	-	-	-
		2	Three roots	0	0	0	0	0	0	0	2	0	-	-	-
Alnaqbi et al. [60]	UAE (local)	54	CBCT	1.9	5.6	5.6	25.9	51.9	3.7	1.9	0	3.7	13.0	83.3	3.7

	UAE (non-local, South Asian)	53		1.9	1.9	0	9.4	58.5	7.5	7.5	0	13.2	3.8	96.2	0
Faraj et al. [8]	IRQ	142	Rx/CR total	0.70	0.70	0	70.43	26.05	0	0	2.12	0	29.57	68.31	2.12
Gündüz & Özlek, [61]	TUR	966	CBCT total	7.6	9.5	8.0	67.8	1.9	0.4	0.5	4.3	0	31.2	64.5	4.3
		446	M	7.2	8.5	6.5	69.7	1.6	0.4	0.2	5.8	0	27.6	66.6	5.8
		520	F	7.9	10.4	9.2	66.2	2.1	0.4	0.8	3.1	0	34.2	62.7	3.1
Hanif et al. [62]	PAK	203	CBCT total	7.9	14.3	1.5	55.2	19.2	1.5	0.5	0	0	11.3	88.7	0
		99	M	8.1	16.2	2	55.6	18.2	0	0	0	0	10.1	89.9	0
		104	F	7.7	12.5	1	54.8	20.2	2.9	1	0	0	12.5	87.5	0
		104	14	7.6	11.5	1.9	56.7	21.2	0.9	0	0	0	-	-	-
		99	24	8.1	17.2	1.0	53.5	17.2	2.0	1.0	0	0	-	-	-
Iqbal et al. [63]	SAU	346	CBCT	58	38.7	1.1	1.1	0.8	0	0	0	0	26.5	72.7	0.5
Khattak et al. [22]	PAK*	250	SC/CR	24.8	8.8	0	30	30.4	0	0	2	4	-	-	-
Medina-Guevara et al. [64]	MEX	841	CBCT total	20.3	13.4	14.9	17.2	31.7	0.4	0	0.1	2.0	48.5	33.9	1.5
		296	M	23.6	12.2	13.1	17.9	29.1	0	0	0.3	3.7	48.6	33.1	2.7
		545	F	18.4	14.2	15.8	16.9	33.3	0.60	0	0.0	0.9	483	34.7	0.2
Olczak et al. [65]	POL	350	CBCT total	1.7	8.6	2.6	78.5	5.1	0	0.6	2.9	-0	28.3	69.1	2.6

		145	M	1.4	6.2	1.4	82.1	4.1	0	0	4.8	0	27.6	68.3	4.1
		205	F	2.0	10.2	3.4	76.1	5.8	0	1.0	1.5	0	28.8	69.7	1.5
		179	14	1.7	7.3	2.2	80.4	4.5	0	0.6	3.3	0	23.5	73.7	2.8
		171	24	1.8	9.9	2.9	76.7	5.8	0	0.6	2.3	0	33.3	64.4	2.3
Peiris et al. [23]	LKA F	71	SC; Mic	1.4	21.1	4.2	57.7	8.5	7	0	0	0	77.5	22.5	0
	LKA M	62		1.6	11.3	0	62.9	4.8	8.1	1.6	0	9.7	66	33.9	0
Allawi et al. [66]	SYR	500	CBCT Total	-	-	-	-	-	-	-	-	-	39.6	59.2	1.2
		198	Single rooted	5.1	41.8	0	25.0	2.0	47.0	0	0	2.0	-	-	-
		296	Two rooted, B	98.7	1.3	0	0	0	0	0	0	0	-	-	-
			Two rooted, P	99.3	0	0.7	0	0	0	0	0	0	-	-	-
		6	Three rooted, MB	100	0	0	0	0	0	0	0	0	-	-	-
			Three rooted, DB	100	0	0	0	0	0	0	0	0	-	-	-
			Three rooted, P	100	0	0	0	0	0	0	0	0	-	-	-
Erkan et al. [67]	TUR	539	CBCT Total	10.2	6.7	3.9	77	0.4	0.4	0	1.5	0	51	49	0
		268	14	10.8	6	5.2	75	0.4	0	0	2.2	0	48.9	51.1	0

		271	24	9.6	7.4	2.6	78.6	0.4	0.4	0	1.5	0	53.1	46.9	0
Khanna et al. [68]	IND*	137	CBCT	8.02	8.7	8.02	65.6	2.9	4.3	0	0	2.1	32.8	67.1	0
Merhej et al. [69]	LBN	480	CBCT Total	8.4	2.7	2.3	80.5	3.3	2.6	0	0.4	0	14.4	85.3	0.4
		240	14	7.1	2.9	2.5	81.3	3.3	2.5	0	0.4	0	13.3	86.3	0.4
		240	24	9.6	2.5	2.1	79.6	3.3	2.5	0	0.4	0	15.4	84.2	0.4
Mirah et al. [70]	SAU	316	14 Total	1.85	12.3	0	83.95	0.95	0	0	0.95	0	15.75	83.3	0.95
		297	24 Total	3	12.6	0	83.4	0	0	0	1	0	17	82	1
Retamoso-Palomino et al. [71]	PER	307	CBCT Total	23.8	11.1	11.1	39.4	5.2	1	3.6	2.9	2	52.1	45	2.9
		152	14	23	9.2	12.5	41.4	2.9	0.7	2.6	3.3	3.3	52	44.7	3.3
		155	24	24.5	12.9	9.7	37.4	6.5	1.3	4.5	2.6	0.6	52.3	45.2	2.6
		133	M	15.8	6.8	6.8	51.1	4.5	0.8	6	5.3	3	-	-	-
		174	F	29.9	14.4	14.4	30.5	5.7	1.1	1.7	1.1	1.1	-	-	-
Shah [72]	PAK*	266	CBCT Total	45.4	3.4	10.9	25.9	12	0.3	0	0	0	92.8	7.14	0
		178	M	48.3	4.5	9.5	23.6	11.2	0	2.2	0.56	0	92.7	7.3	0
		88	F	39.7	1.1	13.6	30.6	13.6	1.1	0	0	0	93.2	6.8	0
Akotiya et al. [73]	IND	360	CBCT	3.3	8.9	7.8	66.7	7.8	0	0	5.6	0	7.8	87.8	4.4
Aljawhar et al. [74]	IRQ	572	CBCT	5.6	0.3	13.8	46.7	16.6	0	8.4	2.4	6.1	47.7	51.1	1.2
		241	M	1.2	0	13.3	51.9	14.9	0	6.6	4.2	7.9	40.7	57.3	2.1
		331	F	8.8	0.6	14.2	42.9	17.8	0	9.7	1.2	4.8	52.9	46.5	0.6

		118	<20 J.	10.2	0.9	12.7	46.6	19.5	0	4.2	0.9	5.1	-	-	-
		135	20-29 J.	8.2	0	7.4	46.7	20.7	0	6.7	3.7	6.7	-	-	-
		115	30-39 J.	5.2	0.9	8.7	53.9	13.9	0	9.6	1.7	6.1	-	-	-
		79	40-49 J.	1.3	0	15.2	45.6	19	0	7.6	5.1	6.3	-	-	-
		68	50-59 J.	1.5	0	17.6	50	13.2	0	14.7	1.5	1.5	-	-	-
		57	>60 J.	1.7	0	35.1	29.8	7	0	12.3	1.7	12.2	-	-	-
Mirza et al. [75]	SAU	656	CBCT	15.68	6.7	1.06	72.9	1.67	0	0	1.67	1.7	31.7	66.61	1.7
		139	M 14	7.3	2.7	0.3	28.9	1.2	0	0	1.2	0.3	-	-	-
		193	M 24	5.9	2.8	0.3	32.5	0.9	0	0	1.2	0.3	-	-	-
		142	F 14	9.3	3.6	0.9	43.4	0.6	0	0	0.3	0	-	-	-
		182	F 24	9	4.3	0.6	41	0.6	0	0	0.6	0	-	-	-
Syed et al. [76]	SAU	399	CBCT Total	7	7	3.3	56.9	24.6	1.3	0	0	0	-	-	-
		192	M	3.1	7.3	3.6	60.9	22.9	2.1	0	0	0	-	-	-
		207	F	10.6	6.8	2.9	53.1	26	0.5	0	0	0	-	-	-
Aljawhar et al. [77]	IRQ	151	CBCT total	2	0	5.3	49.7	11.3	0	12.6	3.3	15.9	45.7	54.3	0
Almehrzi et al. [78]	UAE	355	CBCT total	53.8	10.1	18.3	8.5	9.3	0	0	0	0	52.1	47	0.9
Mustafa et al. [79]	SAU	290	CBCT total	2.76	0	14.8	79.3	1.4	0	0	0.7	1.03	19	79.65	1.38
		190	M	2.1	0	14.20	80	1.57	0	0	0.52	1.57	-	-	-

		100	F	4	0	16.0	78	1	0	0	1.0	0	-	-	-
		8	10-20	0	0	0	100	0	0	0	0	0	-	-	-
		109	> 20-30	2.76	0	11	85.32	0	0	0	0	0	-	-	-
		132	> 30-40	3.78	0	12.12	78.04	3.04	0	0	1.5	1.5	-	-	-
		30	> 40-50	0	0	40.93	58.07	0	0	0	0	0	-	-	-
		9	> 50-60	0	0	11.12	88.88	0	0	0	0	0	-	-	-
		1	> 60-70	0	0	100	0	0	0	0	0	0	-	-	-
Suresh et al. [80]	PAK	67	CBCT total	50.7	10.4	6.0	25.4	6.0	0	0	1.5	0	50.7	47.8	1.5
		36	M	31.3	4.5	1.5	14.9	1.5	0	0	0	0	50	47.2	2.78
		31	F	19.4	6	4.5	10.4	4.5	0	0	1.5	0	51.6	48.4	0
Yanqui-Gómez et al. [81]	PER	392	CBCT total	9.7	11.7	12.5	52	8.2	0.8	2	3.1	0	37	59.9	3.1
		196	M	8.67	6.12	11.73	57.14	9.70	1.02	1.53	4.08	0	-	-	-
		196	F	10.7	17.35	13.27	46.94	6.63	0.51	2.55	2.04	0	-	-	-
		98	20-29	17.3	9.18	13.27	41.84	10.20	2.04	2.04	4.08	0	-	-	-
		98	30-39	2.04	8.16	12.24	61.22	11.22	0	1.02	4.08	0	-	-	-
		98	40-49	3.06	19.38	6.12	60.20	8.16	0	1.02	2.02	0	-	-	-
		98	50-60	3.06	19.39	6.12	60.20	8.16	0	1.02	2.04	0	-	-	-
Jung et al. [82]	KOR	585	CBCT Total	29.1	13.3	9.9	42.6	4.8	0	0.3	0	0	71.45	28.55	0.0
		311	M	26	33	9	48.2	5.5	0	0.6	0	0	-	-	-
		274	F	32.5	16.4	10.9	36.1	4.0	0	0	0	0	-	-	-
		418	Single root	50.7	18.7	13.9	21.8	4.5	0	0.5	0	0	-	-	-

		167	Two roots	0	0	0	94.6	5.4	0	0	0	0	-	-	-
Martins et al. [83]	POR	381	CBCT	3.9	16.3	0	69.3	0.3	4.7	0	0.8	4.7	46.7	50.4	2.9
	JAM	346	CBCT	2.6	0	7.8	0.6	81.5	0	0.6	6.1	0.8	25.7	68.8	5.5
		239	M	-	-	-	-	-	-	-	-	-	25.5	66.1	8.4
		488	F	-	-	-	-	-	-	-	-	-	42.2	55.8	2.0
Acevedo-Tavie et al. [84]	CHL	268	CBCT Total	18.3	13	9	52.2	3.7	1.9	1.5	0.4	0	56.3	43.3	0.4
		87	M	-	-	-	-	-	-	-	-	-	51.7	48.3	0
		181	F	-	-	-	-	-	-	-	-	-	58.5	41	0.5
			Single root	32.5	23.2	15.9	19.2	3.9	3.3	2	0	0	-	-	-
			Two roots	0	0	0	95.7	3.4	0	0.9	0	0	-	-	-
			Three roots	0	0	0	0	0	0	0	100	0	0	0	0
Hafiizh et al. [85]		282	CBCT total	14.9	0	14.2	51.8	12.1	0.7	4.3	0	2.1	65.2	34.8	0
	MYS	205	Total	14.1	0	14.6	54.1	9.3	0.5	4.9	0	2.4	60	40	0
		77	M	15.6	0	7.8	66.2	3.9	1.3	1.3	0	3.9	42.9	57.1	0
		128	F	13.3	0	18.8	46.9	12.5	0	7	0	1.6	70.3	29.7	0
	CHN	66	Total	16.7	0	15.2	40.9	22.7	1.5	3	0	0	81.8	18.2	0
		28	M	10.7	0	17.9	35.7	32.1	0	3.6	0	0	85.7	14.3	0
		38	F	21.1	0	13.2	44.7	15.8	2.6	0	0	57.6	78.9	21.1	0
	IND	11	Total	18.2	0	72.7	0	0	0	0	0	9.1	36.4	63.6	0
		9	M	0	0	88.9	0	0	0	0	0	11.1	22.2	77.8	0
		2	F	100	0	0	0	0	0	0	0	0	100	0	0

Watanabe et al. [86]	JAP	726	CBCT total	12.1	17.5	7	55.5	4.5	1.1	0.6	1.2	0.4	12.1	86.2	1.6
		213	M	7.5	14.6	3.8	66.7	3.3	1.9	0	1.4	0.9	49.8	48.8	1.4
		513	F	14	18.7	8.4	36	5.1	0.8	100	1.2	0.2	71	27.9	1.2
		108	≤20	17.6	13.9	7.4	45.4	12	0.9	0.9	1.9	0	71.3	26.9	1.9
		308	21-40	13.3	14.0	10.1	55.5	4.5	0.6	0.3	0.6	1.4	64.6	34.7	0.6
		207	41-60	8.7	21.7	3.4	58.9	4.5	2.4	0.5	1.4	0	63.8	34.8	1.4
		103	≥61	9.7	23.3	4.9	59.2	0	0	1.0	1.9	0	60.2	37.9	1.9
